# Hepatoprotective Effects of *Citri reticulatae* Pericarpium and *Chaenomelese speciosa* (Sweet) Nakai Extracts in Alcohol-Related Liver Injury: Modulation of Oxidative Stress, Lipid Metabolism, and Gut Microbiota

**DOI:** 10.3390/antiox14030343

**Published:** 2025-03-14

**Authors:** Shuangshuang Ma, Lingtao Kang, Zhipeng Gao, Zhaoping Pan, Lvhong Huang, Jiaxu Chen, Yanfang Liao, Jiajing Guo, Fuhua Fu

**Affiliations:** 1 Longping Branch, College of Biology, Hunan University, Changsha 410125, China; ssma@hnu.edu.cn (S.M.); klt0427@hnu.edu.cn (L.K.); lliao666@hnu.edu.cn (Y.L.); 2 Dongting Laboratory, Hunan Agricultural Product Processing Institute, Hunan Academy of Agricultural Sciences, Changsha 410125, China; pzpjl@163.com (Z.P.); huanglvhong26@163.com (L.H.); chenjiaxu@hunaas.cn (J.C.); 3 College of Fisheries, Hunan Agricultural University, Changsha 410128, China; gaozhipeng627@163.com

**Keywords:** *Citri reticulatae* pericarpium, *Chaenomelese speciosa* (Sweet) Nakai, alcohol-related liver injury, gut microbiota, transcriptome

## Abstract

Chronic and excessive alcohol consumption induces alcohol-related liver injury (ALI), characterized by oxidative stress (OS), disrupted lipid metabolism, and gut microbiota dysbiosis. Given the lack of effective pharmacological treatments, flavonoid-rich fruits have attracted growing attention as potential intervention strategies. This study investigated the independent and combined effects of extracts from *Citri reticulatae* pericarpium (CRPE) and *Chaenomeles speciosa* (Sweet) Nakai (CSPE), previously shown to possess hepatoprotective properties, in a mouse model of ethanol-induced chronic ALI. The flavonoid composition of CRPE and CSPE was characterized using LC-MS/MS, and their potential mechanisms of action were further elucidated through transcriptomic analysis. The results showed that CRPE and CSPE, whether administered individually or in combination, effectively alleviated alcohol-induced hepatic histological damage and inflammatory responses. Furthermore, both extracts significantly reduced OS and improved lipid metabolism. Notably, CRPE, CSPE, and their combination regulated the gut microbiota, as shown by increased abundances of beneficial bacteria such as *Lactobacillus* and *Bifidobacterium*, along with elevated levels of short-chain fatty acids (SCFAs). These findings highlight that combinations of multiple fruit extracts exhibit significant potential in alleviating ALI by modulating the gut microbiota, providing valuable insights for the development of functional foods.

## 1. Introduction

Alcohol-related liver injury (ALI) is a serious and increasingly common liver disorder driven by chronic and excessive alcohol consumption [1]. Over the past few years, the incidence of ALI has risen sharply, contributing significantly to the global burden of liver-disease-related morbidity and mortality, while placing considerable strain on healthcare systems and economies [2,3,4]. The pathogenesis and progression of ALI involve a complex and dynamic pathological process, with oxidative stress (OS) recognized as a central driving factor. During alcohol metabolism, the excessive production of reactive oxygen species (ROS) leads to lipid peroxidation (LPO), DNA damage, and inactivation of antioxidant enzymes, culminating in hepatocyte injury [5,6]. Moreover, alcohol disrupts lipid metabolism, resulting in abnormal fat accumulation, which exacerbates the progression of fatty liver disease [7]. The gut–liver axis is central to the initiation and progression of ALI. The gut–liver axis represents a bidirectional communication system linking the gut and liver, facilitated by the bile ducts, portal vein, and systemic circulation [8]. As an essential component of the gut–liver axis, the gut microbiota plays a key role in the pathogenesis of ALI [9,10,11]. Research has demonstrated that alcohol consumption disrupts the homeostasis of the gut microbiota, resulting in decreased levels of short-chain fatty acids (SCFAs), which in turn compromise liver function [12,13]. Nevertheless, conventional pharmacological treatments for ALI, including disulfiram, naltrexone, acamprosate, and nalmefene, while partially effective, are frequently accompanied by notable side effects [14]. Consequently, the identification of safer and more sustainable therapeutic options has emerged as a critical priority in contemporary research.

Flavonoids are bioactive secondary metabolites derived from edible and medicinal homologous plants. Flavonoids have garnered considerable interest in clinical practice owing to their favorable safety profile and multifunctional properties. Studies have shown that flavonoids ameliorate ALI through diverse mechanisms, including enhancing ethanol metabolism, modulating OS, suppressing inflammatory responses, improving lipid metabolism, and preventing hepatocyte apoptosis [15,16]. Fruits, as a rich dietary source of flavonoids, are not only integral to a balanced diet but also represent a natural strategy for preventing and managing ALI. *Citri reticulatae* pericarpium (CRP) and *Chaenomeles speciosa* (Sweet) Nakai (CSP) are representative plant resources rich in flavonoids. CRP demonstrates a broad spectrum of biological activities, such as antioxidant, anti-inflammatory, anticancer, and lipid-regulating properties [17]. The primary flavonoids in CRP, including hesperidin, nobiletin, and tangeretin, have been shown to mitigate liver injury caused by various factors [18]. CSP is renowned for its antibacterial and antioxidant properties, alongside its ability to inhibit pancreatic lipase and α-amylase [19,20]. However, the mechanisms through which ethanol extracts of *Citri reticulatae* pericarpium (CRPE) and *Chaenomeles speciosa* (Sweet) Nakai (CSPE) alleviate alcohol-driven hepatic OS and lipid metabolism disorders remain largely unexplored, especially in relation to their impact on gut microbiota modulation. Therefore, our hypothesis is based on the hepatoprotective effect and underlying mechanisms of flavonoid-rich CRPE and CSPE, suggesting that they may attenuate the initiation and development of ALI by enhancing antioxidant defenses and modulating gut microbiota composition.

This study aims to systematically investigate the hepatoprotective effects and molecular mechanisms of CRPE and CSPE, administered individually or in combination, in a mouse model of ALI. By integrating histopathological assessment, biochemical analysis, gut microbiota profiling, and transcriptomic analysis, this study elucidates the potential mechanisms through which CRPE and CSPE mitigate ALI. These findings highlight their role as functional food components and provide a theoretical basis for the development of natural intervention strategies targeting ALI. Additionally, this work establishes a foundation for future research into the application of functional foods in preventing and managing alcohol-related liver disorders.

## 2. Materials and Methods

### 2.1. Preparation of CRPE and CSPE

CRP and CSP were sourced from Jiangmen City, Guangdong Province, and Baoshan City, Yunnan Province, respectively. CRP and CSP were ground into powder, and CRPE and CSPE were prepared using the ultrasound-assisted extraction (UAE) method. The extraction process was performed in an ultrasonic water bath. Samples were mixed with a 60% ethanol–water solution at a solid-to-liquid ratio of 1:30 (*w*/*v*) and extracted for 1 h. The extract was centrifuged at 8000 rpm for 15 min at 4 °C to collect the supernatant. This centrifugation process was repeated three times to ensure thorough extraction. The collected supernatants were combined and filtered through a 0.45 μm polyethersulfone (PES) aqueous filter membrane. The resulting filtrate was vacuum-concentrated at 50 °C using a rotary evaporator. The concentrated solution was freeze-dried under vacuum for 48 h to obtain the lyophilized CRPE and CSPE, which were subsequently stored at −20 °C for further experiments.

### 2.2. Analysis of Active Ingredients in CRPE and CSPE

One hundred milligrams CRPE and CSPE were separately added to 500 μL of 80% methanol aqueous solution. Subsequently, centrifugation was performed at 15,000× *g* for 20 min at 4 °C, and the supernatant was collected and diluted to a methanol content of 53%. After a second round of centrifugation, the supernatant was collected for LC-MS/MS analysis. Sample separation was performed using a Vanquish UHPLC system (Thermo Fisher, Dreieich, Germany) equipped with a Hypersil Gold column (100 × 2.1 mm, 1.9 μm, Thermo Fisher, Waltham, MA, USA). The detection was performed with a Q Exactive™ HF mass spectrometer (Thermo Fisher, Dreieich, Germany). Mobile phase A consisted of 0.1% formic acid aqueous solution (Thermo Fisher, Waltham, MA, USA), while mobile phase B was methanol (Thermo Fisher, Waltham, MA, USA). The flow rate was set at 0.2 mL/min, and the column temperature was maintained at 40 °C. The chromatographic gradient elution program was defined as follows: 0–1.5 min, 98% A; 1.5–3 min, 15% A; 3–10 min, 0% A; and 10–12 min, 98% A. The mass spectrometer was operated with a scan range of a mass-to-charge ratio (*m*/*z*) of 100–1500. Data processing was conducted using a Linux operating system (CentOS version 6.6).

### 2.3. Determination of the In Vitro Antioxidant Activity of CRPE and CSPE

The hydroxyl radical scavenging activity and total antioxidant capacity (2,2-Diphenyl-1-picrylhydrazyl (DPPH) radical scavenging test and the 2,2′-Azinobis (3-ethylbenzothiazoline-6-sulfonic acid) (ABTS) radical scavenging test) of the samples were determined using the microplate method, following the instructions provided by the commercial kits (Suzhou Comin Biotechnology Co., Ltd., Suzhou, China). The scavenging capacities of hydroxyl radicals, DPPH radicals, and ABTS radicals were measured at 510 nm, 515 nm, and 734 nm, respectively, using a microplate reader (SYNERGY H1, Santa Clara, CA, USA).

### 2.4. Animal Experiment

Thirty-six male C57BL/6J mice (6–8 weeks old, 20 ± 2 g) were obtained from Hunan SJA Laboratory Animal Co., Ltd. (Changsha, China) and maintained under specific pathogen-free conditions. The housing environment was controlled with a 12 h light/dark cycle, a temperature of 22 ± 2 °C, and a relative humidity of 60% ± 10%. During the experimental period, the mice were provided ad libitum access to sterilized feed and autoclaved water. Following a one-week acclimation period, the mice were randomly divided into six groups (*n* = 6 per group): (1) a normal control group (CON), (2) an alcohol model group (MOD), (3) a positive control group (silybin, SIB), (4) a CRPE intervention group (CRPE), (5) a CSPE intervention group (CSPE), and (6) a CRPE and CSPE combined intervention group (C+C, 1:1 ratio). All extracts were dissolved in distilled water. The mice were administered daily gavage treatments, consisting of extract doses of 200 mg/kg·BW/d or SIB doses of 60 mg/kg·BW/d, in a total volume of 200 μL. The CON group was administered an equivalent volume of distilled water. One hour after administration, all groups, except for the CON group, were gavaged with ethanol solution (10 mL/kg·BW/d). In contrast, the CON group was given with an equivalent volume of distilled water. During the first week of the experiment, the ethanol concentration was gradually increased from 5% (*v*/*v*) to 50% (*v*/*v*). The experiment was conducted over 8 weeks, with mouse body weight recorded weekly. All animal care and experimental procedures were approved by the Biomedical Research Ethics Committee of the Hunan Academy of Agricultural Sciences (no. 2023012).

### 2.5. Euthanasia and Biomaterial Collection

At the end of the experiment, mice were subjected to overnight fasting (water was provided ad libitum). Blood samples were obtained through retro-orbital bleeding, followed by centrifugation to isolate serum for biochemical analysis. Mice were then euthanized by cervical dislocation. Following euthanasia, the liver was immediately dissected and weighed. Liver and colonic tissues from the same anatomical region were fixed in a 4% paraformaldehyde solution (Solarbio, Beijing, China) for histological evaluation. The remaining liver tissues were rapidly frozen in liquid nitrogen and stored at −80 °C for subsequent analyses.

### 2.6. Histological Analysis

The liver tissues from mice were thoroughly rinsed with physiological saline and then fixed in 4% paraformaldehyde solution for 24 h. The fixed samples were subjected to a series of processing steps, including dehydration, clearing, and paraffin embedding. The paraffin-embedded tissues were sectioned into 4-μm-thick slices and subsequently stained with hematoxylin and eosin (H&E). The H&E-stained pathological sections were examined and characterized using a light microscope at 100× magnification (Primostar 3, ZEISS, Oberkochen, Germany). A portion of the samples was preserved at −80 °C, sectioned using a cryostat, and stained with Oil Red O. Oil Red O-stained sections were scanned and subsequently observed and analyzed using the SlideViewer software (v2.6) at 100× magnification.

### 2.7. Biochemical Analysis

Serum levels of aspartate aminotransferase (AST), alanine aminotransferase (ALT), triglycerides (TG), and total cholesterol (TC) were quantified using enzyme-linked immunosorbent assay (ELISA) kits. Approximately 100 mg of liver tissue was homogenized in 1000 μL of PBS buffer to prepare a tissue homogenate. The homogenate was then centrifuged at 10,000 rpm for 10 min at 4 °C, and the supernatant was carefully collected. Liver biochemical parameters, including AST, ALT, TG, TC, superoxide dismutase (SOD), malondialdehyde (MDA), glutathione peroxidase (GSH-Px), catalase (CAT), interleukin-6 (IL-6), and tumor necrosis factor-α (TNF-α) were assessed. All parameters were quantified using commercially available ELISA kits (Shanghai Kexing Trading Co., Ltd., Shanghai, China), and quantitative analyses were performed at 450 nm with a microplate reader following the manufacturer’s instructions.

### 2.8. SCFAs Analysis of Fecal Content

Fresh fecal samples from mice were collected and promptly stored at −80 °C until further analysis. A 20 mg fecal sample was accurately weighed and mixed with 800 μL of 0.5% phosphoric acid solution containing 10 μg/mL of the internal standard 2-ethylbutyric acid. The samples were homogenized using a cryogenic grinder operating at 20 Hz for 3 min, followed by ultrasonic disruption for 10 min. The homogenized samples were then centrifuged at 13,000× *g* for 15 min at 4 °C to separate the supernatant. A 200 μL aliquot of the supernatant was carefully collected and combined with an equal volume (200 μL) of n-butanol to perform solvent extraction. The mixture was vortexed for 10 s, followed by another round of ultrasonic disruption and centrifugation. The final supernatant was collected for subsequent analysis. The quantification of SCFAs was performed using an 8890B-5977B GC/MSD system (Agilent Technologies Inc., Santa Clara, CA, USA) equipped with an HP-FFAP capillary column and high-purity helium as the carrier gas. A split injection was performed at 180 °C with a split ratio of 10:1 and an injection volume of 1 μL. The column temperature was initially set to 80 °C, ramped at 20 °C/min to 120 °C, then further increased at 5 °C/min to 160 °C, and finally held at 220 °C for 3 min.

### 2.9. 16S rRNA Gene Sequencing and Microbiome Analysis

Total genomic DNA was extracted from mouse fecal samples using the E.Z.N.A. Stool DNA Kit (Omega Bio-tek, Norcross, GA, USA) following the manufacturer’s instructions. The quality of the extracted DNA was assessed by 1% agarose gel electrophoresis. The V3–V4 hypervariable regions of the 16S rRNA gene were amplified by PCR using the universal primers 338F (5′-ACTCCTACGGGAGGCAGCAG-3′) and 806R (5′-GGACTACHVGGGTWTCTAAT-3′). The amplified PCR products were subsequently used to construct sequencing libraries. Sequencing was performed on the Illumina NextSeq 2000 platform by Majorbio Bio-Pharm Technology Co., Ltd. (Shanghai, China). Raw paired-end reads were first quality-filtered using fastp (version 0.19.6) and then merged with FLASH (version 1.2.11). The merged sequences were then clustered into operational taxonomic units (OTUs) and chimera-checked using UPARSE (version 7.1). The taxonomic classification of representative OTU sequences was conducted using the RDP classifier (version 2.11) with the Silva 16S rRNA gene database (version 138).

### 2.10. Transcriptional Analysis

Total RNA was extracted from mouse liver tissue samples using the TRIzol reagent (Life Technologies, Inc., South San Francisco, CA, USA) according to the manufacturer’s instructions. The concentration and purity of the RNA were measured using a NanoDrop 2000 spectrophotometer (Thermo Fisher Scientific, Waltham, MA, USA). RNA integrity was evaluated by agarose gel electrophoresis, and the RNA Quality Number (RQN) was determined using the Agilent 5300 (Agilent, Santa Clara, CA, USA) system. Library construction was performed using SMART-Seq_V4 Ultra Low Input RNA Kit for Sequencing (Clontech, San Diego, CA, USA), followed by high-throughput sequencing on the NovaSeq X Plus platform (Illumina, San Diego, CA, USA). Raw sequencing data were filtered using the fastp software (https://github.com/OpenGene/fastp, accessed on 11 June 2024) to obtain high-quality clean data, and quantification analysis was conducted using the RSEM software (http://deweylab.github.io/RSEM/, accessed on 11 June 2024). RNA sequencing and subsequent data analysis were performed by Majorbio Bio-Pharm Technology Co., Ltd. (Shanghai, China).

### 2.11. Statistical Analysis

Statistical analyses were conducted using IBM SPSS Statistics (v27.0), and data visualization was performed with GraphPad Prism 8.0. All data are expressed as the mean ± standard deviation. Multiple group comparisons were performed using one-way analysis of variance (ANOVA), followed by Tukey’s post hoc test. Statistical significance was defined as *p* < 0.05. Hierarchical clustering analysis and principal coordinate analysis (PCoA) were performed based on unweighted UniFrac distances. The significance of differences in the microbiome and transcriptome data was assessed using the Kruskal–Wallis H test, followed by Dunn’s post hoc test for further pairwise comparisons.

## 3. Results

### 3.1. Chemical Characteristics of CRPE and CSPE

A comprehensive phytochemical analysis of CRPE and CSPE was conducted using LC-MS/MS. The compounds in both extracts were characterized under both positive- and negative-ion modes. CRPE was found to contain 964 compounds in total, including 226 phenolic compounds; 53 terpenoids; 91 fatty acyls; 79 amino acids, peptides, and their analogs; 18 steroids and their derivatives; 27 alkaloids and their derivatives; and 13 organic nitrogen compounds. Among the 226 phenolic compounds, 63 were O-methylated flavonoids, 57 were flavonoid glycosides, 27 were isoflavones, 13 were flavones, and 12 were flavans. In contrast, CSPE was found to contain 749 compounds, including 126 phenolic compounds; 71 terpenoids; 54 fatty acyls; 72 amino acids, peptides, and their analogs; 15 steroids and their derivatives; 8 alkaloids and their derivatives; and 12 organic nitrogen compounds. Among the 126 phenolic compounds, 31 were O-methylated flavonoids, 27 were isoflavones, 22 were flavonoid glycosides, 8 were flavans, and 5 were flavones. Phenolic compounds, particularly flavonoids, are well recognized as major contributors to the antioxidant activity in natural products, primarily through mechanisms such as free radical scavenging and the inhibition of LPO [21]. Consequently, the analysis of chemical composition differences between CRPE and CSPE primarily focused on the types and the relative abundances of flavonoids. Further analysis identified significant differences in the types and the relative abundances of flavonoids between CRPE and CSPE. The relative abundances of compounds were ranked based on their average peak areas derived from the mass spectra. Table 1 lists the top 15 flavonoids in CRPE ranked by their relative abundances, including Hexamethylquercetagetin, 3,3′,4′,5,6,7,8-Heptamethoxyflavone, Isosinensetin, Nobiletin, Salvigenin, 5-O-Demethylnobiletin, 5-Desmethylsinensetin, Chrysosplenetin, Juglanin, 4′,5,7-Trimethoxyflavone, 9,10-Dihydro-8-hydroxy-10-methyl-8H-pyrano[2,3-h]epicatechin, Quercetin 3,5,7,3,4-Pentamethyl ether, Tangeritin, Hesperidin, and Melisimplin. Similarly, Table 2 lists the top 15 flavonoids in CSPE ranked by their relative abundances. These compounds include Catechin, Isosinensetin, 6-Demethoxytangeretin, Morin, 3,3′,4′,5,6,7,8-Heptamethoxyflavone, Nobiletin, Quercimeritrin, 5-O-Demethylnobiletin, Leucocyanidin, 7-Hydroxy-5,6,8,3′-Tetramethoxy-4′,5′-Methylenedioxyflavone, Hexamethylquercetagetin, Kaempferol 3-O-alpha-L-galactoside, Rhodiosin, Epicatechin 5-O-beta-D-glucopyranoside, and 4′-Hydroxyflavanone. Additionally, shared compounds between CRPE and CSPE, including 3,3′,4′,5,6,7,8-Heptamethoxyflavone, Isosinensetin, and Nobiletin, as shown in the tables, may make important contributions to the antioxidant activity of both extracts. Meanwhile, compounds unique to each extract may be the key factors driving the differences in their efficiency in scavenging various free radicals.

### 3.2. In Vitro Antioxidant Properties of CRPE and CSPE

**Figure 1:**
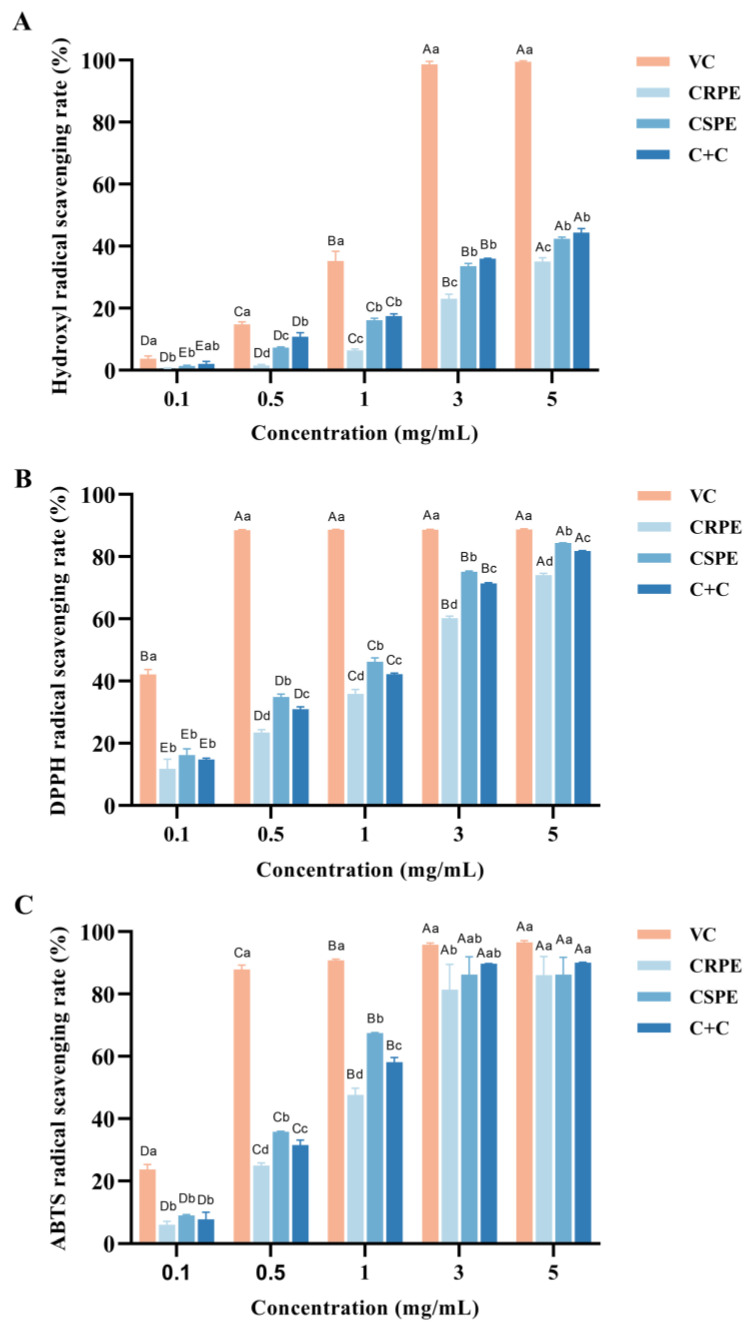
In vitro antioxidant properties of CRPE, CSPE and C+C. (**A**) Hydroxyl radical scavenging rate; (**B**) DPPH radical scavenging rate; (**C**) ABTS radical scavenging rate. Bars with different uppercase letters indicate significant differences between groups (*p* < 0.05), while bars with different lowercase letters indicate significant differences within groups (*p* < 0.05).

The antioxidant activities of CRPR and CSPE, both individually and in combination, were evaluated using hydroxyl radical (·OH), DPPH, and ABTS radical scavenging assays. As shown in Figure 1, within the concentration range of 0.1–5 mg/mL, the hydroxyl radical scavenging rate of VC increased from 3.69 ± 0.77% to 99.49 ± 0.24%, the DPPH radical scavenging rate increased from 42.09 ± 1.32% to 88.69 ± 0.25%, and the ABTS radical scavenging rate increased from 23.70 ± 1.33% to 96.51 ± 0.49%. The antioxidant capacities of CRPE, CSPE, and C+C increased significantly with increasing concentrations, and the differences among the three gradually diminished. At the concentration of 5.0 mg/mL, the DPPH radical scavenging rates for CRPE, CSPE, and C+C were 74.02 ± 0.50%, 84.41 ± 0.06%, and 81.78 ± 0.15%, respectively, while their ABTS radical scavenging rates reached 86.01 ± 4.90%, 86.17 ± 4.58%, and 90.01 ± 0.17%, which were comparable to the antioxidant activity of VC. Notably, CSPE exhibited the highest DPPH radical scavenging activity. Furthermore, C+C demonstrated superior hydroxyl radical and ABTS radical scavenging abilities compared to CRPE and CSPE, suggesting that its higher diversity of flavonoid compounds may contribute to enhanced antioxidant capacity. This result suggests that the interaction among compounds in the C+C group may have contributed to the enhancement of its antioxidant capacity; however, the specific mechanisms require further investigation.

### 3.3. CRPE and CSPE Alleviated ALI in Mice

**Figure 2:**
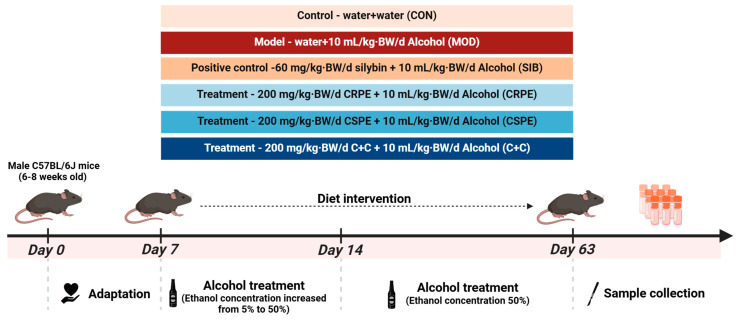
Experimental design of the animal study.

**Figure 3:**
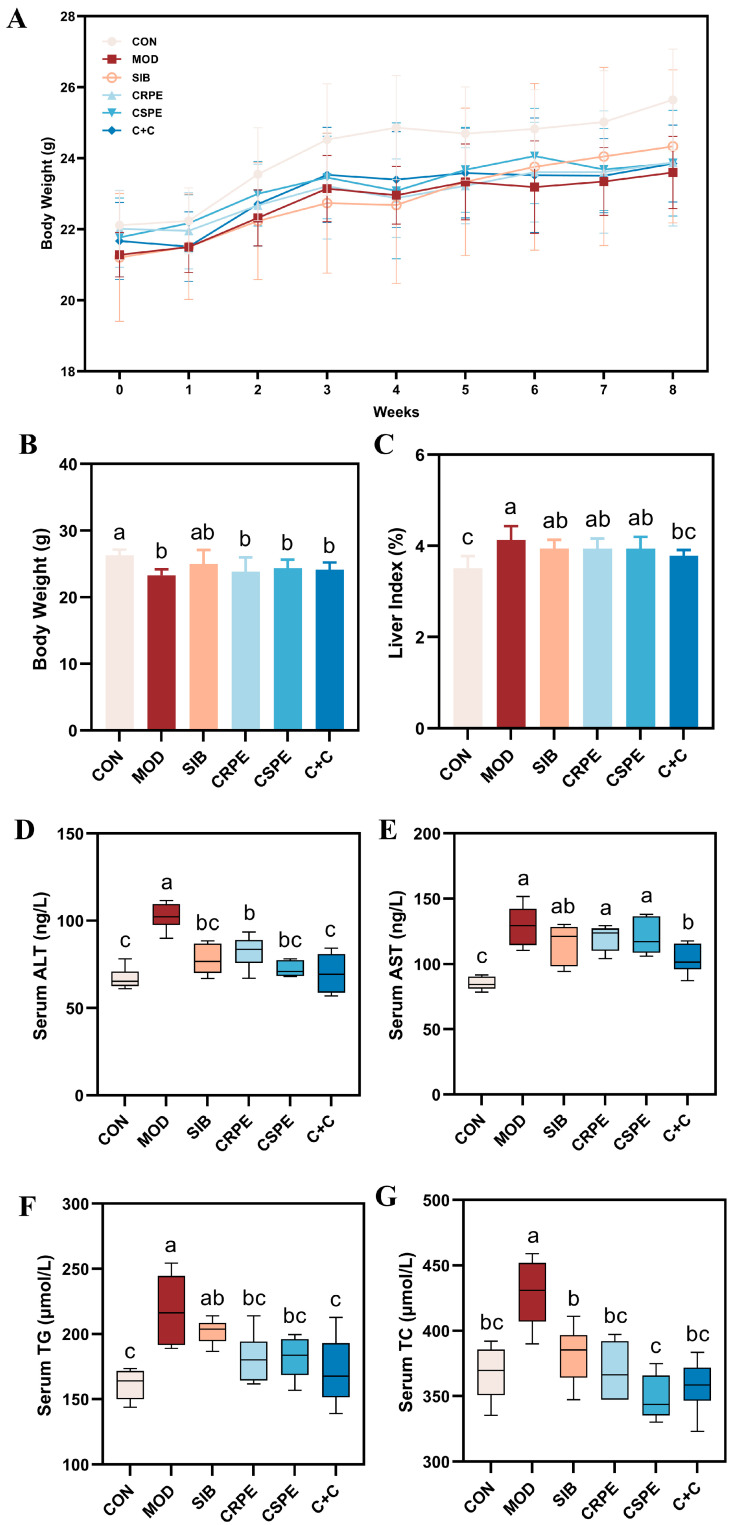
CRPE, CSPE, and C+C alleviated ALI in mice. (**A**) Changes in body weight during the experiment; (**B**) final body weight at the end of the experiment; (**C**) liver-to-body weight ratio at the end of the experiment; (**D**) serum ALT activity; (**E**) serum AST activity; (**F**) serum TG levels; (**G**) serum TC levels. Bars with different lowercase letters indicate significant differences between groups (*p* < 0.05).

We examined the effects of CRPE, CSPE, and C+C on chronic ALI through an eight-week dietary intervention study in mice (Figure 2). Throughout the experiment, all mice remained in good health. The monitoring of body weight (Figure 3A) demonstrated that alcohol consumption significantly impaired weight gain in mice. By the conclusion of the experiment, the body weight of mice in the MOD group was significantly lower than that of the CON group, whereas the CRPE, CSPE, and C+C interventions partially alleviated this weight loss (Figure 3B). Furthermore, the liver-to-body-weight ratio was significantly elevated in the MOD group (Figure 3C), whereas interventions with CRPE, CSPE, and C+C markedly reduced the liver index. The liver index in the C+C group was the lowest, suggesting that this combination of dietary supplements may exert a more pronounced effect in preventing pathological hepatomegaly.

Regarding biochemical parameters, serum AST and ALT levels, recognized as sensitive markers of liver injury, were markedly elevated in the MOD group (Figure 3D,E) [22]. In contrast, interventions with SIB and the extracts significantly attenuated AST and ALT activity. Notably, among all intervention groups, the C+C treatment group exhibited the most significant reduction in AST and ALT levels, indicating its superior hepatoprotective effects. Additionally, excessive alcohol consumption typically resulted in lipid metabolism disorders, characterized by significantly elevated levels of TG and TC in the serum (Figure 3F,G). Supplementation with SIB, CRPE, CSPE, and C+C partially ameliorated these metabolic abnormalities, significantly reducing serum TG and TC levels. Additionally, the C+C group demonstrated the most significant effect in reducing serum TG levels, further highlighting its superior ability to alleviate alcohol-driven metabolic dysfunction. These findings indicated that SIB and the extracts effectively alleviated ALI in mice.

**Figure 4:**
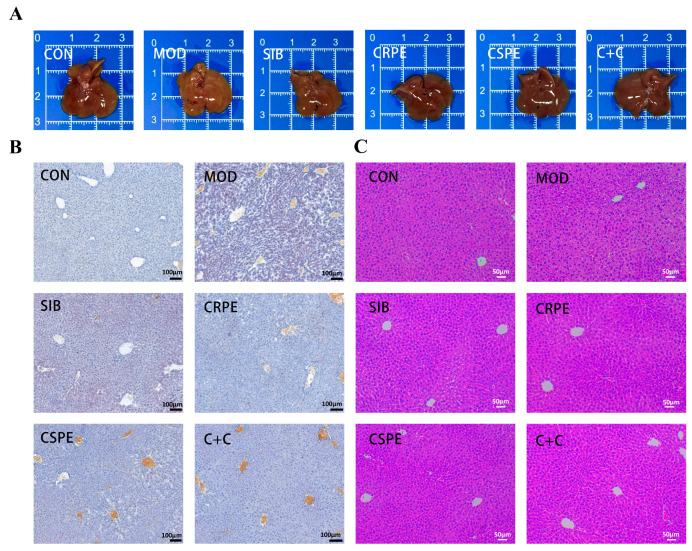
CRPE, CSPE, and C+C alleviated pathological liver tissue damage in ALI mice. (**A**) Representative photographs of mouse livers in each group; (**B**) Oil Red O-stained liver sections for each group; (**C**) H&E-stained liver sections for each group.

Morphological observations (Figure 4A) showed that the livers of MOD group mice displayed abnormal coloration and a loss of characteristic glossiness, whereas those in the CRPE, CSPE, and C+C groups retained a normal bright red appearance. Histopathological analysis examination further confirmed the protective effects of CRPE, CSPE, and C+C on the liver. Liver tissues from the MOD group exhibited significant lipid accumulation, as evidenced by Oil Red O staining (Figure 4B). Interventions with SIB, CRPE, CSPE, and C+C markedly reduced hepatic lipid accumulation. H&E staining (Figure 4C) revealed that alcohol exposure disrupted hepatocyte structure, leading to disorganized cell arrangement, sparse cytoplasm, and widespread tissue damage. In contrast, the CRPE, CSPE, and C+C interventions significantly restored hepatocyte structural integrity and normalized cell arrangement. These findings suggested that interventions with CRPE, CSPE, and C+C effectively mitigated alcohol-mediated liver damage in mice.

### 3.4. CRPE and CSPE Alleviated OS and Inflammation Triggered by Alcohol in Mouse Liver

**Figure 5:**
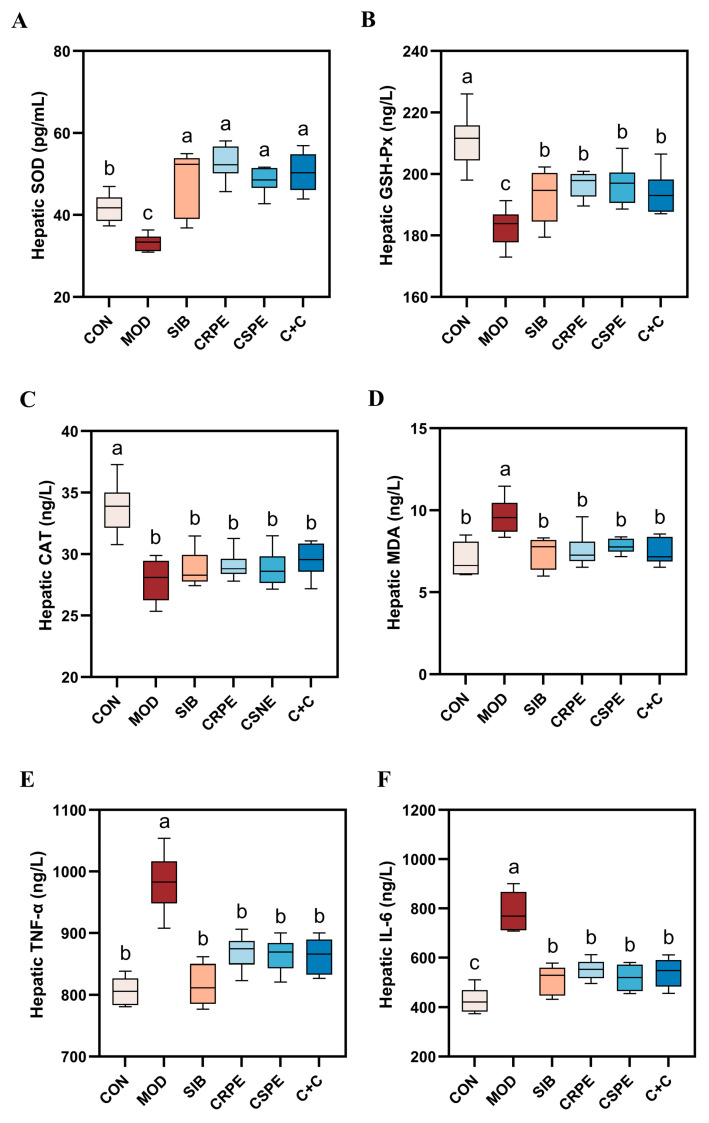
CRPE, CSPE, and C+C alleviated alcohol-induced OS and inflammation in mice. (**A**) Hepatic SOD levels; (**B**) hepatic GSH-Px levels; (**C**) hepatic CAT levels; (**D**) hepatic MDA levels; (**E**) hepatic TNF-α levels; (**F**) hepatic IL-6 levels. Bars with different lowercase letters indicate significant differences between groups (*p* < 0.05).

To evaluate the impact of CRPE, CSPE, and C+C on alcohol-driven OS and inflammatory responses, OS markers and pro-inflammatory cytokines were quantified. Alcohol metabolism generates substantial amounts of ROS. While the endogenous antioxidant defense system neutralizes some ROS, excessive ROS deplete endogenous antioxidants, impairing the body’s overall antioxidant capacity. This imbalance triggers OS, leading to cellular and tissue damage. SOD, GSH-Px, and CAT represent indispensable antioxidant enzymes responsible for scavenging superoxide anions, with their activities reflecting tissue antioxidant capacity. The experimental results demonstrated that alcohol exposure significantly diminished the activities of SOD, GSH-Px, and CAT, indicating a pronounced elevation in OS levels in the livers of mice with chronic liver injury. However, interventions with SIB, CRPE, CSPE, and C+C significantly restored the activities of these antioxidant enzymes to levels close to normal (Figure 5A–C). Importantly, the C+C group exhibited the highest CAT activity among all intervention groups, suggesting its superior capacity to counteract OS. Furthermore, malondialdehyde (MDA), a critical marker of LPO, was significantly elevated in the liver tissues of the MOD group. Treatments with SIB, CRPE, CSPE, and C+C notably reduced MDA levels (Figure 5D), with the C+C group showing the most pronounced reduction. These findings indicated that the C+C extract might have possessed a greater ability to suppress LPO and enhance antioxidant defenses.

An analysis of inflammatory markers revealed that the levels of key pro-inflammatory cytokines, IL-6 and TNF-α, were significantly elevated in the liver tissues of the MOD group compared to the CON group (Figure 5E,F), indicating that chronic alcohol consumption intensifies hepatic inflammation. However, interventions with CRPE, CSPE, and C+C significantly reduced IL-6 and TNF-α levels in comparison to the MOD group, with the C+C group demonstrating the most pronounced reduction in TNF-α. These findings indicated that the extracts effectively alleviated OS and inflammatory responses in mice with ALI.

### 3.5. CRPE and CSPE Increase the Production of SCFAs

**Figure 6:**
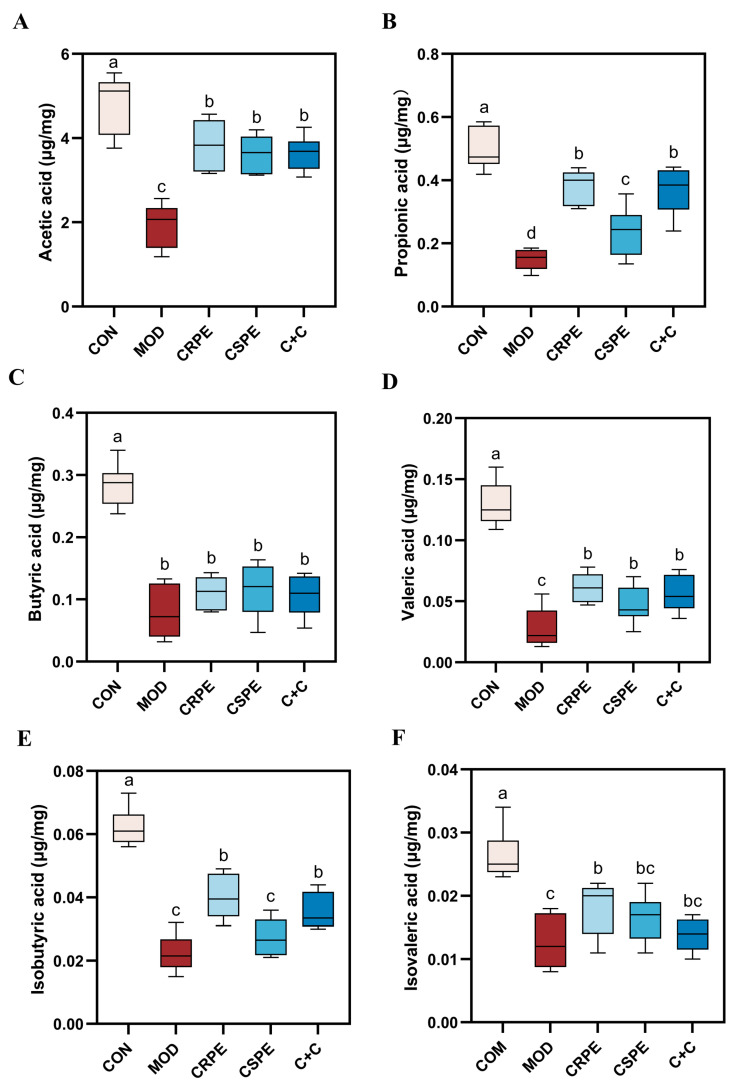
CRPE, CSPE, and C+C increase the production of SCFAs. (**A**) Acetic acid; (**B**) propionic acid; (**C**) butyric acid; (**D**) valeric acid; (**E**) isobutyric acid; (**F**) isovaleric acid. Bars with different lowercase letters indicate significant differences between groups (*p* < 0.05).

SCFAs, which are essential metabolites produced through gut microbial fermentation, are pivotal in maintaining intestinal barrier integrity and systemic immune homeostasis in the host [23]. To further investigate this, we measured SCFAs levels in mouse fecal samples. The results indicated that the concentrations of acetic acid, propionic acid, butyric acid, valeric acid, isobutyric acid, and isovaleric acid were significantly reduced in the MOD group compared to the CON group (Figure 6A–F), demonstrating that alcohol consumption adversely affected SCFA metabolism. In contrast, treatments with CRPE, CSPE, and C+C significantly increased the levels of these SCFAs compared to the MOD group, although no statistically significant differences were detected among the treatment groups. Notably, CRPE demonstrated superior efficacy in enhancing SCFAs production, while the C+C combination showed greater improvement compared to CSPE alone. These findings indicate that CRPE significantly boosted the gut microbiota’s capacity to produce SCFAs, which may underlie mitigating effects on ALI.

### 3.6. CRPE and CSPE Altered the Structure and Composition of Gut Microflora

**Figure 7:**
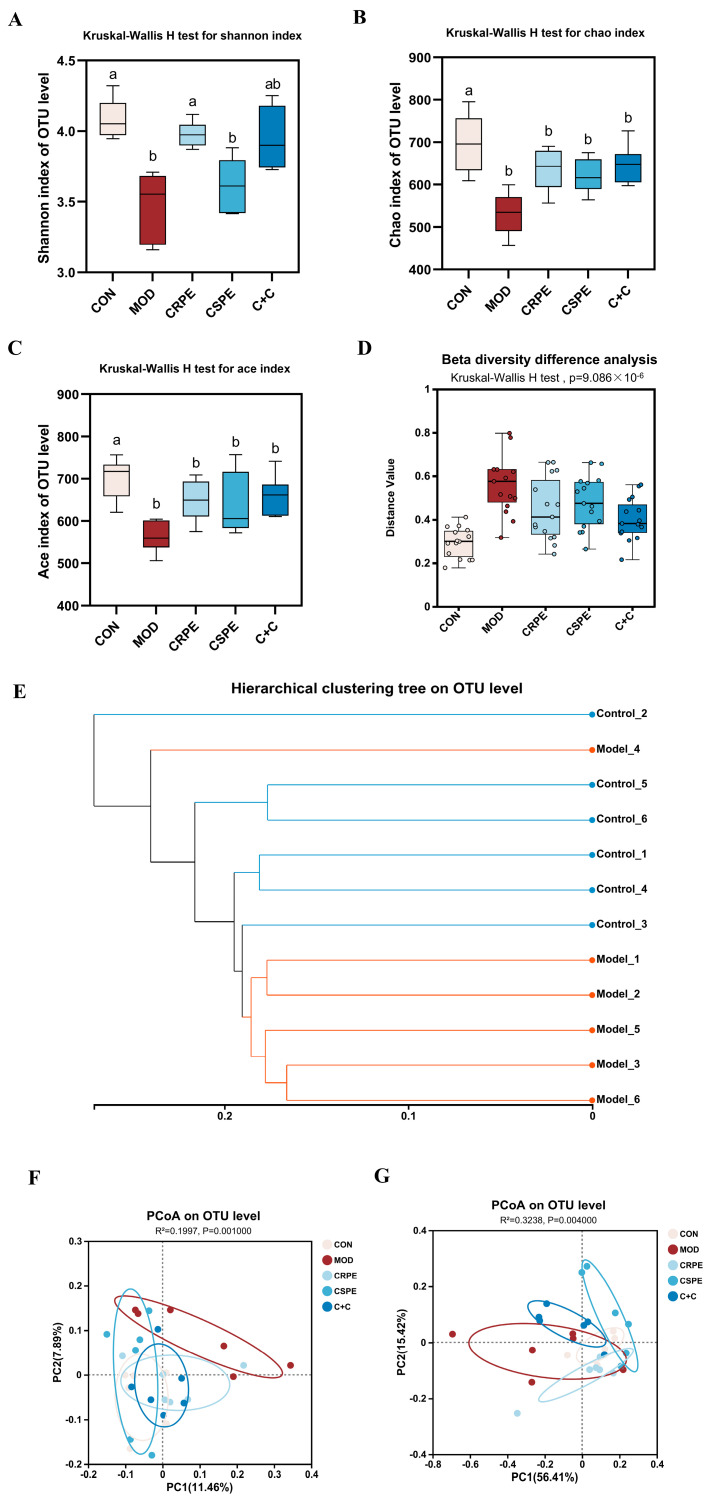
Effects of CRPE, CSPE, and C+C on the diversity of the gut microbiota in ALI mice. (**A**–**C**) Alpha diversity indices at the OTU level; Bars with different lowercase letters indicate significant differences between groups (*p* < 0.05). (**D**) beta diversity analysis at the OTU level; (**E**) hierarchical clustering analysis at the OTU level; (**F**) PCoA of gut microbiota composition at the OTU level based on unweighted UniFrac distances; (**G**) PCoA of gut microbiota composition at the OTU level based on weighted UniFrac distances.

This study employed 16S rRNA gene amplicon sequencing to explore the potential regulatory effects of CRPE, CSPE, and C+C on gut microbiota in mice with ALI. At the OTU level, alpha diversity was analyzed (Figure 7A–C). The results demonstrated that alcohol-fed mice exhibited a significant reduction in microbial diversity, as indicated by decreased Chao and ACE indices (indicating community richness) and Shannon index (indicating community diversity), suggesting that chronic alcohol consumption likely leads to a substantial decline in gut microbiota diversity [24]. Following supplementation with CRPE, CSPE, and C+C, all indices exhibited a trend toward recovery, although changes in Chao and ACE indices were not statistically significant. These findings suggest that CRPE, CSPE, and C+C interventions may ameliorate the alcohol-induced decline in gut microbiota richness and evenness. Further beta diversity analyses, including intergroup significance testing (Figure 7D) and hierarchical clustering analysis (Figure 7E), revealed significant differences in the bacterial community structure at the OTU level between the MOD and CON groups. The unweighted UniFrac distance analysis, which accounts for the presence or absence of microbial taxa, underscored notable differences in community composition. PCoA based on unweighted UniFrac distances (Figure 7F) demonstrated that CRPE, CSPE, and C+C interventions significantly modulated the gut microbiota structure, aligning it more closely with the composition observed in the CON group. In contrast, the weighted UniFrac distance analysis, integrating both the presence and relative abundance of taxa, offered a more comprehensive view of community composition. PCoA based on weighted UniFrac distances (Figure 7G) similarly revealed significant intergroup differences, further supporting the regulatory effects of CRPE, CSPE, and C+C on the gut microbial community structure.

**Figure 8:**
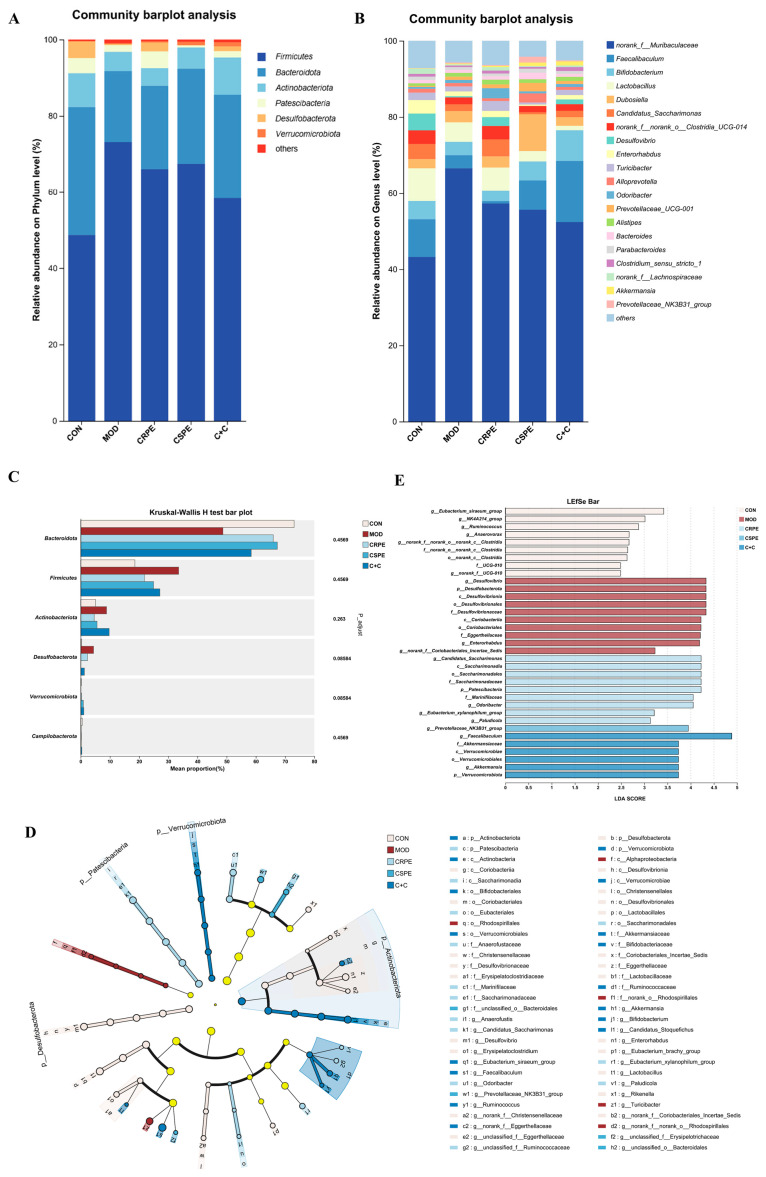
Effects of CRPE, CSPE, and C+C on the structure and composition of the gut microbiota in ALI mice. (**A**) Taxonomic composition of the gut microbiota at the phylum level; (**B**) taxonomic composition of the gut microbiota at the genus level; (**C**) intergroup differential significance testing of taxa; (**D**) cladogram representing the taxonomic distribution among groups determined by LEfSe analysis; (**E**) linear discriminant analysis effect size (LEfSe, LDA score > 2).

To gain a comprehensive understanding of gut microbiota alterations, bacterial taxonomic similarities were examined in greater detail at both the phylum and genus levels. At the phylum level (Figure 8A), *Firmicutes*, *Bacteroidetes*, and *Actinobacteriota* were identified as the dominant phyla. Chronic alcohol consumption, compared to the CON group, led to an increased relative abundance of *Firmicutes* and disrupted the *Firmicutes*-to-*Bacteroidetes* ratio (F/B), which is widely recognized as a hallmark of alcohol-induced gut microbiota dysbiosis [23]. Notably, interventions with CRPE, CSPE, and C+C effectively counteracted these adverse changes, though the observed differences did not achieve statistical significance. At the genus level (Figure 8B), a total of 20 genera were identified, revealing notable shifts in the distribution of dominant taxa among the different groups (Figure S1). CRPE, CSPE, and C+C interventions significantly enhanced the abundance of *Akkermansia* and *Parabacteroides*, two genera intimately linked to improved gut health. Moreover, distinct regulatory effects on specific genera varied across the treatment groups. Specifically, CRPE notably elevated the abundance of *Lactobacillus*, *Bacteroides*, *Prevotellaceae_UCG-001*, and *Candidatus Saccharimonas*; CSPE significantly increased *Faecalibaculum*, *Bifidobacterium*, *Prevotellaceae_UCG-001*, and *Dubosiella*; while C+C predominantly enhanced *Faecalibaculum* and *Bifidobacterium*. Microbial taxa exhibiting significant intergroup differences were identified via the Kruskal–Wallis rank-sum test (Figure 8C). The results showed that the CRPE, CSPE, and C+C interventions markedly reduced the abundance of *Desulfobacterota* while substantially increasing that of *Verrucomicrobiota*, providing further evidence that these treatments effectively modulated alcohol-induced gut microbiota dysbiosis [25]. To characterize microbial taxa exhibiting significant differences in more depth, LEfSe analysis was performed using a linear discriminant analysis (LDA) threshold set at 2.0 (Figure 8D). The results indicated that CRPE and CSPE treatments significantly enriched probiotic taxa, including *Bifidobacterium* and *Akkermansia*, implying that these treatments may enhance gut health by modulating the gut microbiota composition. The bacterial taxa enriched by the C+C treatment were similar to those in the CRPE and CSPE groups, but with a higher enrichment magnitude, indicating that the combined treatment may exert a stronger synergistic effect on restoring gut microbial homeostasis. Further analysis of LDA scores (Figure 8E) revealed that the highest-ranked taxa were *Candidatus Saccharimonas*, *Prevotellaceae_NK3B31_group*, and *Faecalibaculum* in the CRPE, CSPE, and C+C groups, respectively.

### 3.7. Potential Mechanisms of CRPE and CSPE in Alleviating ALI Identified by Transcriptome Analysis

**Figure 9:**
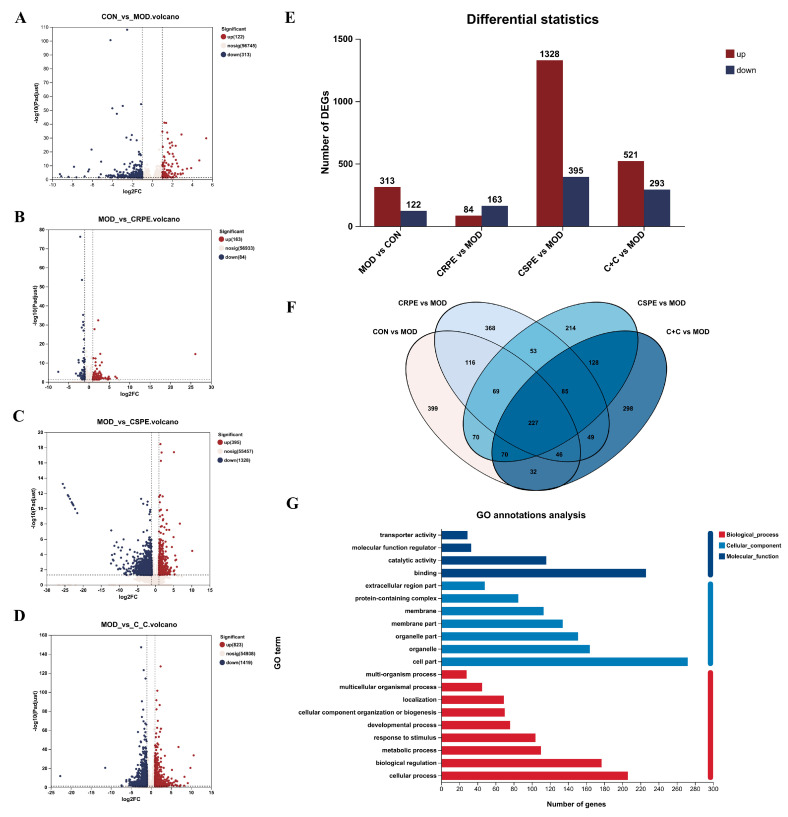

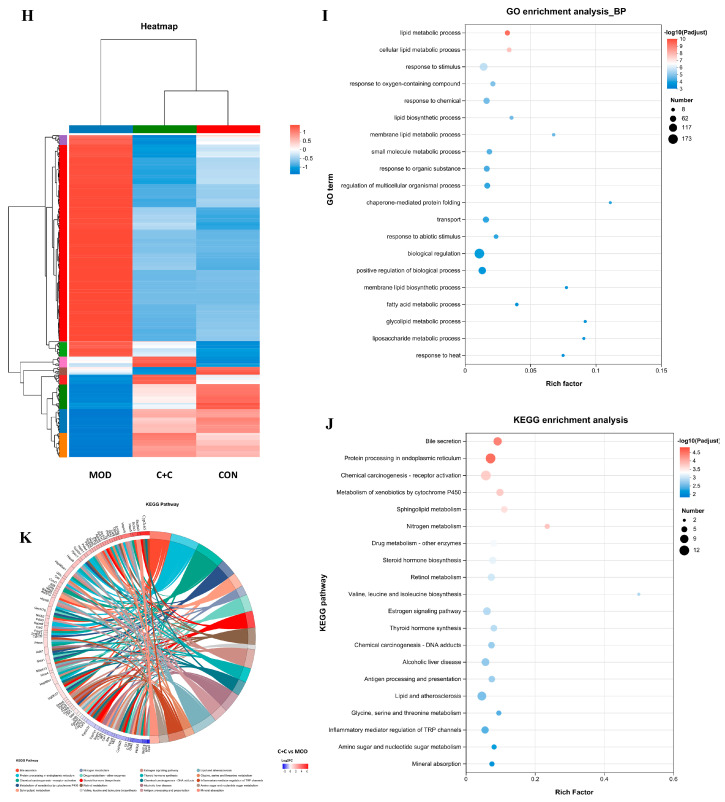
Transcriptomic analysis of mRNA in the liver tissues of ALI mice treated with CRPE, CSPE, and C+C. (**A**–**D**) Volcano plots of DEGs; (**E**) bar graph of DEGs; (**F**) Venn diagram of DEGs shared among groups; (**G**) KEGG pathway annotation analysis of DEGs; (**H**) heatmap of shared DEGs generated by hierarchical clustering analysis; (**I**) GO enrichment analysis of DEGs in biological processes (BP).; (**J**) KEGG pathway enrichment analysis of DEGs; (**K**) KEGG pathway enrichment chord diagram of DEGs in C+C vs. MOD group.

Transcriptome analyses were performed to investigate the potential mechanisms by which CRPE, CSPE, and C+C mitigated ALI. The differentially expressed genes (DEGs) identified between groups were visualized through volcano plots (Figure 9A–D). As illustrated in Figure 9E, 435 DEGs in total were identified in the MOD group when compared with the CON group, including 313 upregulated and 122 downregulated genes. Compared to the MOD group, 247, 1723, and 814 DEGs were identified in the CRPE, CSPE, and C+C groups, respectively. Specifically, 84, 1328, and 521 upregulated genes, along with 163, 395, and 293 downregulated genes, were detected in the CRPE, CSPE, and C+C groups, respectively. The analysis of specific DEG distributions revealed 399, 368, 214, and 298 unique DEGs in the CON, CRPE, CSPE, and C+C groups, respectively, when compared to the MOD group. To further investigate the protective effects of C+C on ALI, a total of 375 overlapping DEGs were identified in the comparison between the C+C vs. MOD group and the CON vs. MOD group (Figure S2), indicating that C+C might mitigate ALI by regulating these DEGs (Figure 9F). Gene Ontology (GO) enrichment analysis of these DEGs (Figure 9G) revealed that DEGs related to biological processes were primarily enriched in cellular processes (GO:0009987), biological regulation (GO:0065007), metabolic processes (GO:0008152), and response to stimulus (GO:0050896). Regarding cellular components and molecular functions, these DEGs were predominantly enriched in the cell part (GO:0044464) and binding (GO:0005488) categories, respectively. The hierarchical clustering of the 375 overlapping DEGs (Figure 9H) revealed detailed gene expression patterns. The gene expression profile of the C+C group was more similar to that of the CON group but exhibited significant differences from the MOD group, suggesting that C+C exerted a strong regulatory influence on alcohol-induced DEGs.

To investigate the potential mechanisms underlying the effects of C+C on ALI, GO biological process enrichment analysis was conducted on the 375 DEGs (Figure 9I). The findings indicated that these DEGs were predominantly enriched in lipid metabolic processes, including the lipid metabolic process (GO:0006629), the cellular lipid metabolic process (GO:0044255), the membrane lipid metabolic process (GO:0046467), and the fatty acid metabolic process (GO:0006631). They were also significantly enriched in stress response pathways, such as response to oxygen-containing compounds (GO:1901700) and response to chemicals (GO:0042221), as well as biological regulation processes (GO:0065007). Among these, lipid metabolic processes, particularly lipid metabolic process (GO:0006629) and cellular lipid metabolic process (GO:0044255), exhibited the highest levels of enrichment and statistical significance, indicating that C+C may exert its effects by modulating lipid synthesis and metabolism. Furthermore, a substantial number of genes were enriched in biological regulation (GO:0065007) and the positive regulation of biological processes (GO:0048518), underscoring the significant impact of C+C on overall biological regulatory functions. The considerable enrichment of genes associated with response to stimuli further supports the hypothesis that C+C protects against ALI by regulating alcohol-induced OS responses. Taken together, these findings indicate that the protective effects of C+C against ALI were primarily mediated through its multifaceted regulation of lipid metabolism, OS, and inflammation, consistent with the biochemical assay results. To further elucidate the potential mechanisms by which C+C ameliorated ALI, Kyoto Encyclopedia of Genes and Genomes (KEGG) pathway analysis was conducted (Figure 9J). The analysis identified Bile secretion (Pathway ID: mmu04976) *and* Protein processing in the endoplasmic reticulum (Pathway ID: mmu04141) as the two most significantly enriched pathways, suggesting that C+C exerted its protective effects by enhancing bile metabolism and alleviating endoplasmic reticulum stress. Additionally, pathways associated with lipid metabolism, such as Sphingolipid metabolism (Pathway ID: mmu00600) and Lipid and atherosclerosis (Pathway ID: mmu05417), were significantly enriched, indicating that C+C improves ALI by regulating lipid metabolism and inflammatory responses. Notably, the enrichment of the alcoholic liver disease (Pathway ID: mmu04936) pathway highlighted the potential of C+C to mitigate alcoholic liver disease through multifaceted regulatory mechanisms. Furthermore, the regulation of the cytochrome P450 system (Pathway ID: mmu00980) by C+C likely contributed to reducing the toxicity associated with alcohol metabolism.

This study employed chord diagrams to visualize the effects of C+C treatment on gene expression in ALI, facilitating the analysis of complex gene–pathway interactions (Figure 9K) (Table S1). C+C treatment significantly upregulated the *Abcb1a*, *Apoa4*, *Slc51b*, *Fkbp4*, *Gpx2*, *Gsta1*, HSP family, and *Pgm2* genes, while markedly downregulating *Fkbp5*, compared with the MOD group. These differentially expressed genes are primarily associated with lipid metabolism, inflammation and immune regulation, bile acid metabolism, glycolysis, and OS, suggesting that C+C may mitigate ALI progression through multifaceted regulatory mechanisms.

## 4. Discussion

Chronic and excessive alcohol consumption triggers various physiological changes, such as hepatic OS, disrupted lipid metabolism, and gut microbiota imbalance, which collectively drive the onset and progression of ALI [26]. In response to this issue, flavonoid-rich fruits have attracted substantial attention as potential dietary interventions. *CRP* and *CSP*, two medicinal and edible plants abundant in flavonoids, have been widely investigated for their diverse pharmacological properties. Previous studies have shown that both *CRP* and *CSP* possess hepatoprotective effects to varying degrees. The objective of this research is to investigate the protective effects of CRPE and CSPE, either individually or in combination (C+C), against chronic ALI and to elucidate the underlying mechanisms.

In this study, LC-MS/MS was employed for the qualitative analysis of chemical components in CRPE and CSPE. The results revealed that both extracts are rich in polyphenolic compounds, particularly flavonoids, which are widely regarded as the primary contributors to the antioxidant activity of plant extracts [27]. Although quantitative analysis was not conducted, the LC-MS/MS results suggest that differences in the types of flavonoids between CRPE and CSPE may partially account for the variations in their antioxidant activities. However, the precise mechanisms underlying these differences require further investigation through advanced chemical separation, quantitative analysis, and biological validation experiments. Furthermore, the antioxidant activity may also be influenced by the interactions among various phytochemical constituents within the extracts.

The antioxidant capacities of CRPE, CSPE, and their combination (C+C) were evaluated in vitro by assessing their ability to scavenge hydroxyl radicals, DPPH radicals, and ABTS radicals. The results demonstrated that the antioxidant activities of CRPE, CSPE, and C+C increased significantly with concentration (*p* < 0.05), and the differences among the three tended to diminish at higher concentrations. Notably, C+C exhibited superior scavenging activity for hydroxyl radicals and ABTS radicals compared to CRPE and CSPE, indicating enhanced antioxidant performance. This suggests that the combined extract (C+C) may exhibit synergistic or additive effects, thereby amplifying its antioxidant capacity [28,29].

Chronic alcohol consumption disrupts the hepatic balance between fatty acid catabolism and anabolism, resulting in abnormal lipid accumulation in hepatocytes, a hallmark of ALI [30,31]. Consistent with previous findings, our study revealed that chronic alcohol exposure induces lipid droplet accumulation and structural damage in liver tissues, as confirmed by histological analysis, H&E staining, and Oil Red O staining [32,33]. These results further support the notion that prolonged alcohol consumption disrupts lipid homeostasis, promotes hepatic steatosis, and ultimately leads to liver dysfunction [34]. This study established a chronic ALI mouse model to systematically evaluate the therapeutic effects of CRPE, CSPE, and their combined extract (C+C). Treatment with CRPE, CSPE, and C+C significantly improved liver morphology, attenuated hepatic steatosis, and effectively alleviated overall liver injury compared with the MOD group. These results align with previous studies and further validate the mechanism by which plant-derived polyphenols and flavonoids mitigate ALI through the regulation of lipid metabolism [35]. ALT and AST activities are widely regarded as key biochemical markers of hepatocyte injury, while serum TG and TC levels are critical indicators of lipid metabolism disorders [36,37]. In this study, chronic alcohol consumption significantly increased ALT and AST activities, as well as serum TG and TC levels, consistent with previous findings [38]. Treatment with CRPE, CSPE, and C+C significantly reduced ALT and AST activities in the liver, indicating the hepatoprotective effects of these extracts. Moreover, CRPE, CSPE, and C+C treatments significantly reduced alcohol-induced elevations in serum TG and TC levels, highlighting their role in restoring lipid metabolism balance. These findings further support the efficacy of plant extracts in restoring lipid homeostasis in models of alcohol-induced liver injury. Notably, C+C exhibited significantly greater efficacy in reducing AST, ALT, and TG levels compared with CRPE or CSPE alone, suggesting potential synergistic effects between CRPE and CSPE in protecting hepatocytes and regulating triglyceride metabolism. This finding is consistent with previous studies reporting that the combined use of quercetin and catechin exhibits enhanced antioxidant, anti-inflammatory, and lipid-regulating effects compared to single extracts [39]. The potential synergistic effects may result from complementary interactions among distinct phytochemical constituents [28].

The excessive production of ROS during alcohol metabolism is widely recognized as a critical factor in liver injury [40]. ROS are generated through various pathways, including ethanol metabolism by alcohol dehydrogenase, the activation of cytochrome P450 2E1 (CYP2E1), and mitochondrial dysfunction. These pathways collectively contribute to OS and hepatocellular damage [41]. OS depletes endogenous antioxidant enzymes and disrupts the balance between oxidative and antioxidative defense systems, resulting in LPO and tissue damage [42,43]. Furthermore, ROS activate redox-sensitive pro-inflammatory signaling pathways, further exacerbating inflammation and tissue damage in the liver [44]. This study demonstrates that CRPE, CSPE, and C+C significantly enhance the activities of key antioxidant enzymes, such as SOD, CAT, and GSH-Px. These enzymes are essential for neutralizing ROS and maintaining redox homeostasis. This finding aligns with previous research showing that flavonoid-rich plant extracts and representative flavonoid monomers possess strong antioxidant activity [33]. Furthermore, CRPE, CSPE, and C+C significantly decrease the levels of MDA, a key byproduct of LPO, further supporting their effectiveness in inhibiting LPO [41]. CRPE, CSPE, and C+C markedly suppress the expression of pro-inflammatory cytokines, including TNF-α and IL-1β, in liver tissues, indicating robust anti-inflammatory effects. This anti-inflammatory effect aligns with previous research showing that natural plant extracts effectively reduce the production of pro-inflammatory cytokines in ALI models [25]. In conclusion, this study underscores the antioxidant and anti-inflammatory properties of CRPE, CSPE, and C+C in mitigating ALI.

Recent studies have highlighted the pivotal role of the gut–liver axis in the development and progression of alcoholic liver disease, with gut microbiota dysbiosis recognized as a hallmark feature of ALI [13]. Alcohol and its metabolites perturb the homeostasis of the gut microbiome, promoting the overgrowth of pathogenic bacteria and the depletion of commensal bacteria [45]. High-throughput sequencing in this study identified significant alterations in gut microbiota composition between the MOD and CON groups, consistent with prior findings in human studies. Specifically, individuals with alcoholic fatty liver disease displayed a pronounced decrease in *Bacteroidetes* and *Prevotellaceae*, accompanied by a notable increase in *Firmicutes* and *Proteobacteria* [46]. Moreover, *Firmicutes* and *Bacteroidetes* constitute the two dominant phyla in the human gut microbiota, with an increased abundance of *Firmicutes* strongly correlated with lipid accumulation and elevated TG levels [47,48]. This study demonstrated that CRPE, CSPE, and C+C effectively modulate the gut microbiota by restoring the richness and diversity of gut flora disrupted by alcohol exposure. These treatments significantly reversed the alcohol-induced overgrowth of *Firmicutes* and the elevated F/B ratio. However, the regulatory effects of CRPE and CSPE on the gut microbiota differed, with each treatment targeting distinct microbial taxa. Further analysis revealed that these extracts modulate gut microbiota associated with lipid metabolism, effectively alleviating alcohol-induced elevations in TG and TC levels. Moreover, CRPE, CSPE, and C+C treatments increased the abundance of gut bacteria known to produce SCFAs, including *Lactobacillus*, *Bacteroides*, and *Prevotellaceae*. These changes significantly elevated fecal SCFAs levels, such as acetate, propionate, butyrate, valerate, isobutyrate, and isovalerate [49]. In summary, these findings underscore the critical role of gut microbiota modulation in mitigating alcohol-induced liver injury, highlighting the potential of CRPE, CSPE, and C+C as effective therapeutic agents for restoring gut microbial balance and improving lipid metabolism.

This study conducted a systematic transcriptomic analysis to elucidate the potential mechanisms underlying C+C intervention in ALI. Functional annotation and enrichment analyses were conducted on 375 DEGs regulated by C+C. GO functional annotation revealed that C+C may alleviate ALI by regulating lipid metabolism, stress responses, and biological processes. The hierarchical clustering analysis of DEGs showed that the gene expression profile of the C+C group closely resembled that of the CRPE group, suggesting CRPE’s potential dominant role in the combined treatment. Further GO enrichment analysis revealed that these 375 overlapping DEGs were predominantly enriched in pathways associated with lipid metabolism, stress responses, protein regulation, and biological modulation. These findings underscore the role of C+C in alleviating alcohol-induced liver pathology by regulating lipid metabolism and OS signaling pathways [50]. Moreover, KEGG pathway analysis further corroborated these findings, indicating that C+C mitigates liver injury by regulating endoplasmic reticulum stress, lipid metabolism pathways, and directly targeting the alcoholic liver disease pathway [51,52]. The enrichment chord diagram suggests that C+C treatment may mitigate ALI by regulating bile acid metabolism, lipid metabolism, OS, inflammatory responses, and glycolysis-related pathways. Among the differentially expressed genes, the upregulation of *Abcb1a*, *Apoa4*, *Slc51b*, *Gpx2*, *Gsta1*, and *Hsp90aa1* may be essential for hepatic protection and metabolic homeostasis, while the downregulation of *Fkbp5* may help suppress inflammatory responses and slow ALI progression. Lipid metabolism and bile acid regulation are critical for maintaining hepatic homeostasis and preventing disease. *Abcb1a*, primarily expressed in the liver, small intestine, and kidneys, plays a key role in intestinal drug absorption and bile acid excretion [53]. Its upregulation may promote bile acid secretion, decrease hepatic cholesterol accumulation, and thereby alleviate alcohol-related liver disease-induced hepatic steatosis [54]. Furthermore, *Apoa4*, primarily synthesized in the liver and small intestine, regulates cholesterol transport, facilitates lipoprotein metabolism, and decreases hepatic lipid deposition, while also exerting anti-atherosclerotic and anti-diabetic effects [55,56]. Maintaining bile acid homeostasis is essential for optimal liver function. Slc51b, in conjunction with *Slc51a*, constitutes a bile acid transport system that facilitates bile acid transfer from hepatocytes to systemic circulation. This process decreases intracellular bile acid accumulation, thereby maintaining bile acid homeostasis [57]. In addition to lipid metabolism, OS plays a crucial role in ALI progression. *Gpx2*, an antioxidant enzyme primarily expressed in the liver and intestines, is upregulated in response to OS, enhancing antioxidant defenses and increasing resistance to alcohol-induced oxidative damage [58]. Furthermore, *Gsta1*, a member of the glutathione S-transferase (GST) family, is crucial for reducing LPO and inhibiting ferroptosis [59]. The upregulation of these OS-related genes may protect against hepatocellular damage and strengthen antioxidant defenses. Furthermore, inflammation is a key driver of ALD progression. *Hsp90aa1* may regulate inflammatory responses, cell survival, and stress adaptation, thereby influencing ALI progression [60]. Meanwhile, *Pgm2*, a key enzyme in the glycolysis pathway, participates in glucose metabolism, and its upregulation may support hepatic energy homeostasis [61].

Despite the important findings of this study, several limitations should be acknowledged. First, this study is based on plant extracts, whose chemical composition can vary significantly depending on cultivation conditions, harvest timing, and extraction methods. While the chemical constituents of the extracts were identified and their relative abundances determined via LC-MS/MS, the exact concentrations of individual compounds were not quantified. This limitation hinders a more comprehensive understanding of the dose–effect relationships of the individual compounds. Secondly, although the results indicate that flavonoids exhibit high relative abundance in the extracts and may play a certain role, we have not yet conducted experimental validation on purified flavonoids or their mixtures. Moreover, the complex and dynamic interactions between the gut microbiota and the host are influenced by various environmental factors, adding another layer of complexity to the analysis. Future studies should focus on quantifying the key compounds in plant extracts and conducting functional validation using purified flavonoids and their mixtures. Additionally, systematic studies utilizing multi-omics approaches and dynamic monitoring are required to uncover the specific roles and mechanisms of fruit-derived flavonoids in alleviating ALI.

## 5. Conclusions

To summarize, this study characterized the chemical compositions of CRPE and CSPE, assessed their in vitro antioxidant activities, and demonstrated their pronounced hepatoprotective effects against ALI. Using a chronic ALI mouse model, our experimental results revealed that CRPE, CSPE, and their combination (C+C) effectively alleviated OS and inflammation caused by alcohol. Additionally, our results demonstrate that they effectively mitigate physiological dysfunction and dyslipidemia associated with ALI by regulating triglyceride and cholesterol metabolism, thereby protecting hepatocytes. Furthermore, both individual and combined treatments with CRPE and CSPE modulated the structure of the intestinal microbiome, restored intestinal microbial homeostasis, and increased levels of SCFAs. The transcriptomic analysis further demonstrated that CRPE, CSPE, and C+C mitigated alcohol-induced liver damage predominantly by modulating lipid metabolism and OS-associated signaling pathways. These results highlight the potential of flavonoid-rich CRPE and CSPE as functional ingredients for mitigating ALI, offering compelling scientific evidence for the health benefits of natural flavonoids.

## Figures and Tables

**Table 1:** Characterization and quantification of the top 15 flavonoids in CRPE based on average peak area.

No.	Formula	Molecular Weight	*m*/*z* ^1^	RT ^2^ (min)	Adduct	Compound	Peak Area
1	C_21_H_22_O_8_	402.13	403.14	7.43	[M + H]^+^	Hexamethylquercetagetin	552,559,349,174.21
2	C_22_H_24_O_9_	432.14	433.15	7.53	[M + H]^+^	3,3′,4′,5,6,7,8-heptamethoxyflavone	83,477,038,443.83
3	C_20_H_20_O_7_	372.12	395.11	7.95	[M + Na]^+^	Isosinensetin	39,400,878,448.19
4	C_21_H_22_O_8_	402.13	425.12	8.07	[M + Na]^+^	Nobiletin	15,332,712,531.77
5	C_18_H_16_O_6_	328.09	329.10	6.72	[M + H]^+^	Salvigenin	10,194,311,275.88
6	C_20_H_20_O_8_	388.12	389.12	7.75	[M + H]^+^	5-O-Demethylnobiletin	6,208,555,256.71
7	C_19_H_18_O_7_	358.11	359.11	8.28	[M + H]^+^	5-Desmethylsinensetin	6,055,111,923.25
8	C_19_H_18_O_8_	374.10	373.09	6.73	[M − H]^−^	Chrysosplenetin	5,720,426,977.83
9	C_20_H_18_O_10_	418.09	417.08	5.87	[M − H]^−^	Juglanin	5,502,584,609.53
10	C_18_H_16_O_5_	312.10	313.11	7.71	[M + H]^+^	4′,5,7-Trimethoxyflavone	4,405,711,889.73
11	C_19_H_20_O_7_	360.12	361.13	6.65	[M + H]^+^	9,10-Dihydro-8-hydroxy-10-methyl-8H-pyrano[2,3-h]epicatechin	4,163,792,751.41
12	C_20_H_20_O_7_	372.12	395.11	7.05	[M+ Na]^+^	Quercetin 3,5,7,3,4-pentamethyl ether	4,098,778,344.09
13	C_20_H_20_O_7_	372.12	373.13	0.55	[M + H]^+^	Tangeritin	3,659,997,786.06
14	C_28_H_34_O_15_	610.19	609.18	5.65	[M − H]^−^	Hesperidin	3,612,723,408.31
15	C_19_H_16_O_8_	372.08	373.09	7.43	[M + H]^+^	Melisimplin	3,481,061,795.95

^1^ *m*/*z*, mass-to-charge ratio; ^2^ RT, retention time. This table is presented in descending order based on the average peak area.

**Table 2:** Characterization and Quantification of the Top 15 Flavonoids in CSPE Based on Average Peak Area.

No.	Formula	Molecular Weight	*m*/*z* ^1^	RT ^2^ (min)	Adduct	Compound	Peak Area
1	C_20_H_20_O_7_	372.12	373.13	6.44	[M + H]^+^	Isosinensetin	5,808,079,464.56
2	C_15_H_14_O_6_	290.08	291.09	5.16	[M + H]^+^	Catechin	5,046,372,923.94
3	C_19_H_18_O_6_	342.11	343.12	6.72	[M + H]^+^	6-Demethoxytangeretin	4,017,371,570.56
4	C_15_H_10_O_7_	302.04	303.05	5.6	[M + H]^+^	Morin	2,915,784,800.70
5	C_22_H_24_O_9_	432.14	433.15	6.81	[M + H]^+^	3,3′,4′,5,6,7,8-heptamethoxyflavone	2,594,448,236.13
6	C_21_H_20_O_12_	464.1	465.1	5.6	[M + H]^+^	Quercimeritrin	1,123,926,158.49
7	C_20_H_20_O_8_	388.12	389.12	7	[M + H]^+^	5-O-Demethylnobiletin	1,078,133,775.42
8	C_21_H_22_O_8_	402.13	425.12	6.73	[M+Na]^+^	Nobiletin	963,811,532.09
9	C_15_H_14_O_7_	306.07	289.07	5	[M + H − H_2_O]^+^	Leucocyanidin	757,660,021.50
10	C_20_H_18_O_9_	402.1	403.1	5.25	[M + H]^+^	7-Hydroxy-5,6,8,3′-tetramethoxy-4′,5′-methylenedioxyflavone	657,489,658.88
11	C_21_H_22_O_8_	402.13	403.14	0.52	[M + H]^+^	Hexamethylquercetagetin	562,041,913.85
12	C_21_H_20_O_11_	448.1	449.11	5.73	[M + H]^+^	Kaempferol 3-O-alpha-L-galactoside	415,045,255.05
13	C_15_H_12_O_3_	240.08	223.08	6.98	[M + H − H_2_O]^+^	4′-Hydroxyflavanone	302,956,914.05
14	C_27_H_30_O_16_	610.15	611.16	5.57	[M + H]^+^	Rhodiosin	291,546,369.06
15	C_21_H_24_O_11_	452.13	453.14	4.91	[M + H]^+^	Epicatechin 5-O-beta-D-glucopyranoside	260,292,890.65

^1^ *m*/*z*, mass-to-charge ratio; ^2^ RT, retention time. This table is presented in descending order based on the average peak area.

## Data Availability

All relevant data and materials from this study are provided in the article and Supplementary Materials. For any additional requests, please contact the corresponding author.

## Supplementary Materials

The following supporting information can be downloaded at: https://www.mdpi.com/article/10.3390/antiox14030343/s1, Figure S1: Pie charts illustrating the species composition and relative abundance of microbial communities. Figure S2: Venn diagram of DEGs shared among C+C vs. MOD and MOD vs. CON. Table S1: KEGG pathway enrichment chord diagram data table of DEGs in C+C vs. MOD Group.
