# Supplementary material for: Hepatoprotective Effects of Citri reticulatae Pericarpium and Chaenomelese speciosa (Sweet) Nakai Extracts in Alcohol-Related Liver Injury: Modulation of Oxidative Stress, Lipid Metabolism, and Gut Microbiota
Source: Antioxidants (Basel). 2025 Mar 14;14(3):343. doi: 10.3390/antiox14030343 (PMC11939523; doi:10.3390/antiox14030343)
Supplement: Supplementary file 1 [file antioxidants-14-00343-s001.zip › Table S1.pdf]

Table S1. KEGG Pathway Enrichment Chord Diagram Data Table of DEGs in C+C vs. MOD Group.

Gene ID	Gene name	KEGG Pathway	Pathway ID	log2fc
ENSMUSG00000067231	Cyp2c65	Steroid hormone biosynthesis	mmu00140	6.400635129
ENSMUSG00000067231	Cyp2c65	Retinol metabolism	mmu00830	6.400635129
ENSMUSG00000067231	Cyp2c65	Chemical carcinogenesis - DNA adducts	mmu05204	6.400635129
ENSMUSG00000067231	Cyp2c65	Inflammatory mediator regulation of TRP channels	mmu04750	6.400635129
ENSMUSG00000001225	Slc26a3	Mineral absorption	mmu04978	6.126665561
ENSMUSG00000036123	Slc9a3	Bile secretion	mmu04976	5.125810271
ENSMUSG00000036123	Slc9a3	Mineral absorption	mmu04978	5.125810271
ENSMUSG00000032278	Paqr5	Chemical carcinogenesis - receptor activation	mmu05207	4.96055904
ENSMUSG00000090877	Hspa1b	Protein processing in endoplasmic reticulum	mmu04141	4.051621925
ENSMUSG00000090877	Hspa1b	Estrogen signaling pathway	mmu04915	4.051621925
ENSMUSG00000090877	Hspa1b	Antigen processing and presentation	mmu04612	4.051621925
ENSMUSG00000090877	Hspa1b	Lipid and atherosclerosis	mmu05417	4.051621925
ENSMUSG00000040808	S100g	Mineral absorption	mmu04978	3.840780944
ENSMUSG00000027068	Dhrs9	Retinol metabolism	mmu00830	3.801570432
ENSMUSG00000028463	Car9	Nitrogen metabolism	mmu00910	3.732493167
ENSMUSG00000042808	Gpx2	Thyroid hormone synthesis	mmu04918	3.246513403
ENSMUSG00000024640	Psat1	Glycine, serine and threonine metabolism	mmu00260	3.076215833
ENSMUSG00000053469	Tg	Thyroid hormone synthesis	mmu04918	2.862096412
ENSMUSG00000053862	Slc51b	Bile secretion	mmu04976	2.834301229
ENSMUSG00000029272	Sult1e1	Steroid hormone biosynthesis	mmu00140	2.758202492
ENSMUSG00000032080	Apoa4	Lipid and atherosclerosis	mmu05417	2.755391213
ENSMUSG00000024411	Aqp4	Bile secretion	mmu04976	2.486670597
ENSMUSG00000020911	Krt19	Estrogen signaling pathway	mmu04915	2.443706037
ENSMUSG00000021263	Degs2	Sphingolipid metabolism	mmu00600	2.094698535
ENSMUSG00000031906	Smpd3	Sphingolipid metabolism	mmu00600	2.085921619
ENSMUSG00000056131	Pgm3	Amino sugar and nucleotide sugar metabolism	mmu00520	1.986548093
ENSMUSG00000025823	Pdia4	Protein processing in endoplasmic reticulum	mmu04141	1.897670666
ENSMUSG00000025823	Pdia4	Thyroid hormone synthesis	mmu04918	1.897670666
ENSMUSG00000029657	Hsph1	Protein processing in endoplasmic reticulum	mmu04141	1.891957369

ENSMUSG00000040703	Cyp2s1	Metabolism of xenobiotics by cytochrome P450	mmu00980	1.813926911
ENSMUSG00000040703	Cyp2s1	Retinol metabolism	mmu00830	1.813926911
ENSMUSG00000032115	Hyou1	Protein processing in endoplasmic reticulum	mmu04141	1.808764097
ENSMUSG00000026864	Hspa5	Protein processing in endoplasmic reticulum	mmu04141	1.792210034
ENSMUSG00000026864	Hspa5	Thyroid hormone synthesis	mmu04918	1.792210034
ENSMUSG00000026864	Hspa5	Antigen processing and presentation	mmu04612	1.792210034
ENSMUSG00000026864	Hspa5	Lipid and atherosclerosis	mmu05417	1.792210034
ENSMUSG00000021270	Hsp90aa1	Protein processing in endoplasmic reticulum	mmu04141	1.679376633
ENSMUSG00000021270	Hsp90aa1	Chemical carcinogenesis - receptor activation	mmu05207	1.679376633
ENSMUSG00000021270	Hsp90aa1	Estrogen signaling pathway	mmu04915	1.679376633
ENSMUSG00000021270	Hsp90aa1	Antigen processing and presentation	mmu04612	1.679376633
ENSMUSG00000021270	Hsp90aa1	Lipid and atherosclerosis	mmu05417	1.679376633
ENSMUSG00000024827	Gldc	Glycine, serine and threonine metabolism	mmu00260	1.670987106
ENSMUSG00000003814	Calr	Protein processing in endoplasmic reticulum	mmu04141	1.622831247
ENSMUSG00000003814	Calr	Antigen processing and presentation	mmu04612	1.622831247
ENSMUSG00000070348	Ccnd1	Chemical carcinogenesis - receptor activation	mmu05207	1.557246869
ENSMUSG00000070348	Ccnd1	Alcoholic liver disease	mmu04936	1.557246869
ENSMUSG00000005148	Klf5	Chemical carcinogenesis - receptor activation	mmu05207	1.5298174
ENSMUSG00000028671	Gale	Amino sugar and nucleotide sugar metabolism	mmu00520	1.484257378
ENSMUSG00000079434	Neu2	Sphingolipid metabolism	mmu00600	1.444057766
ENSMUSG00000017929	B4galt5	Sphingolipid metabolism	mmu00600	1.413348391
ENSMUSG00000040584	Abcb1a	Bile secretion	mmu04976	1.322860283
ENSMUSG00000015656	Hspa8	Protein processing in endoplasmic reticulum	mmu04141	1.286875666
ENSMUSG00000015656	Hspa8	Estrogen signaling pathway	mmu04915	1.286875666
ENSMUSG00000015656	Hspa8	Antigen processing and presentation	mmu04612	1.286875666
ENSMUSG00000015656	Hspa8	Lipid and atherosclerosis	mmu05417	1.286875666
ENSMUSG00000111709	Gm3776	Chemical carcinogenesis - receptor activation	mmu05207	1.238013131
ENSMUSG00000111709	Gm3776	Metabolism of xenobiotics by cytochrome P450	mmu00980	1.238013131
ENSMUSG00000111709	Gm3776	Drug metabolism - other enzymes	mmu00983	1.238013131
ENSMUSG00000111709	Gm3776	Chemical carcinogenesis - DNA adducts	mmu05204	1.238013131
ENSMUSG00000037583	Nr0b2	Bile secretion	mmu04976	1.184145276
ENSMUSG00000027248	Pdia3	Protein processing in endoplasmic reticulum	mmu04141	1.167050055

ENSMUSG00000027248	Pdia3	Antigen processing and presentation	mmu04612	1.167050055
ENSMUSG00000025757	Hspa4l	Protein processing in endoplasmic reticulum	mmu04141	1.145139853
ENSMUSG00000027562	Car2	Bile secretion	mmu04976	1.128117215
ENSMUSG00000027562	Car2	Nitrogen metabolism	mmu00910	1.128117215
ENSMUSG00000037348	Paqr7	Chemical carcinogenesis - receptor activation	mmu05207	1.04273355
ENSMUSG00000004460	Dnajb11	Protein processing in endoplasmic reticulum	mmu04141	1.024750708
ENSMUSG00000052974	Cyp2f2	Metabolism of xenobiotics by cytochrome P450	mmu00980	1.014916835
ENSMUSG00000050965	Prkca	Chemical carcinogenesis - receptor activation	mmu05207	1.011339841
ENSMUSG00000050965	Prkca	Thyroid hormone synthesis	mmu04918	1.011339841
ENSMUSG00000050965	Prkca	Lipid and atherosclerosis	mmu05417	1.011339841
ENSMUSG00000050965	Prkca	Inflammatory mediator regulation of TRP channels	mmu04750	1.011339841
ENSMUSG00000055301	Adh7	Metabolism of xenobiotics by cytochrome P450	mmu00980	0.942287561
ENSMUSG00000055301	Adh7	Retinol metabolism	mmu00830	0.942287561
ENSMUSG00000055301	Adh7	Alcoholic liver disease	mmu04936	0.942287561
ENSMUSG00000074183	Gsta1	Chemical carcinogenesis - receptor activation	mmu05207	0.929484382
ENSMUSG00000074183	Gsta1	Metabolism of xenobiotics by cytochrome P450	mmu00980	0.929484382
ENSMUSG00000074183	Gsta1	Drug metabolism - other enzymes	mmu00983	0.929484382
ENSMUSG00000074183	Gsta1	Chemical carcinogenesis - DNA adducts	mmu05204	0.929484382
ENSMUSG00000004864	Mapk13	Alcoholic liver disease	mmu04936	0.886515567
ENSMUSG00000004864	Mapk13	Lipid and atherosclerosis	mmu05417	0.886515567
ENSMUSG00000004864	Mapk13	Inflammatory mediator regulation of TRP channels	mmu04750	0.886515567
ENSMUSG00000024177	Nme4	Drug metabolism - other enzymes	mmu00983	0.878344101
ENSMUSG00000020048	Hsp90b1	Protein processing in endoplasmic reticulum	mmu04141	0.844658653
ENSMUSG00000020048	Hsp90b1	Chemical carcinogenesis - receptor activation	mmu05207	0.844658653
ENSMUSG00000020048	Hsp90b1	Estrogen signaling pathway	mmu04915	0.844658653
ENSMUSG00000020048	Hsp90b1	Thyroid hormone synthesis	mmu04918	0.844658653
ENSMUSG00000020048	Hsp90b1	Lipid and atherosclerosis	mmu05417	0.844658653
ENSMUSG00000057425	Ugt2b37	Bile secretion	mmu04976	0.820533735
ENSMUSG00000057425	Ugt2b37	Chemical carcinogenesis - receptor activation	mmu05207	0.820533735
ENSMUSG00000057425	Ugt2b37	Metabolism of xenobiotics by cytochrome P450	mmu00980	0.820533735
ENSMUSG00000057425	Ugt2b37	Drug metabolism - other enzymes	mmu00983	0.820533735
ENSMUSG00000057425	Ugt2b37	Steroid hormone biosynthesis	mmu00140	0.820533735

ENSMUSG00000057425	Ugt2b37	Retinol metabolism	mmu00830	0.820533735
ENSMUSG00000057425	Ugt2b37	Chemical carcinogenesis - DNA adducts	mmu05204	0.820533735
ENSMUSG00000030357	Fkbp4	Estrogen signaling pathway	mmu04915	0.7668205
ENSMUSG00000029171	Pgm2	Amino sugar and nucleotide sugar metabolism	mmu00520	0.763024737
ENSMUSG00000028008	Asic5	Inflammatory mediator regulation of TRP channels	mmu04750	0.747756634
ENSMUSG00000029817	Tra2a	Alcoholic liver disease	mmu04936	0.700294429
ENSMUSG00000018507	Trpv2	Inflammatory mediator regulation of TRP channels	mmu04750	0.649377124
ENSMUSG00000022947	Cbr3	Metabolism of xenobiotics by cytochrome P450	mmu00980	0.539438092
ENSMUSG00000029596	Sdsl	Valine, leucine and isoleucine biosynthesis	mmu00290	0.449023166
ENSMUSG00000029596	Sdsl	Glycine, serine and threonine metabolism	mmu00260	0.449023166
ENSMUSG00000024924	Vldlr	Lipid and atherosclerosis	mmu05417	0.147891499
ENSMUSG00000049721	Gal3st1	Sphingolipid metabolism	mmu00600	0.043902939
ENSMUSG00000056973	Ces1d	Drug metabolism - other enzymes	mmu00983	-0.174312937
ENSMUSG00000039519	Cyp7b1	Steroid hormone biosynthesis	mmu00140	-0.323773686
ENSMUSG00000027559	Car3	Nitrogen metabolism	mmu00910	-0.356036933
ENSMUSG00000042248	Cyp2c37	Steroid hormone biosynthesis	mmu00140	-0.60312421
ENSMUSG00000042248	Cyp2c37	Retinol metabolism	mmu00830	-0.60312421
ENSMUSG00000042248	Cyp2c37	Chemical carcinogenesis - DNA adducts	mmu05204	-0.60312421
ENSMUSG00000042248	Cyp2c37	Inflammatory mediator regulation of TRP channels	mmu04750	-0.60312421
ENSMUSG00000028240	Cyp7a1	Bile secretion	mmu04976	-0.633416299
ENSMUSG00000028240	Cyp7a1	Steroid hormone biosynthesis	mmu00140	-0.633416299
ENSMUSG00000020538	Srebf1	Alcoholic liver disease	mmu04936	-0.676088837
ENSMUSG00000036905	C1qb	Alcoholic liver disease	mmu04936	-0.774939804
ENSMUSG00000031765	Mt1	Mineral absorption	mmu04978	-0.833669213
ENSMUSG00000027556	Car1	Nitrogen metabolism	mmu00910	-0.890496462
ENSMUSG00000029597	Sds	Valine, leucine and isoleucine biosynthesis	mmu00290	-0.939157261
ENSMUSG00000029597	Sds	Glycine, serine and threonine metabolism	mmu00260	-0.939157261
ENSMUSG00000024222	Fkbp5	Estrogen signaling pathway	mmu04915	-0.941769008
ENSMUSG00000025877	Hk3	Amino sugar and nucleotide sugar metabolism	mmu00520	-0.96895844
ENSMUSG00000029630	Cyp3a25	Chemical carcinogenesis - receptor activation	mmu05207	-0.975423276
ENSMUSG00000029630	Cyp3a25	Steroid hormone biosynthesis	mmu00140	-0.975423276
ENSMUSG00000029630	Cyp3a25	Retinol metabolism	mmu00830	-0.975423276

ENSMUSG00000029630	Cyp3a25	Chemical carcinogenesis - DNA adducts	mmu05204	-0.975423276
ENSMUSG00000025574	Tk1	Drug metabolism - other enzymes	mmu00983	-1.0356238
ENSMUSG00000036887	C1qa	Alcoholic liver disease	mmu04936	-1.266301119
ENSMUSG00000028467	Gba2	Sphingolipid metabolism	mmu00600	-1.588266516
ENSMUSG00000039936	Pik3cd	Chemical carcinogenesis - receptor activation	mmu05207	-1.707813086
ENSMUSG00000039936	Pik3cd	Estrogen signaling pathway	mmu04915	-1.707813086
ENSMUSG00000039936	Pik3cd	Lipid and atherosclerosis	mmu05417	-1.707813086
ENSMUSG00000039936	Pik3cd	Inflammatory mediator regulation of TRP channels	mmu04750	-1.707813086
ENSMUSG00000035699	Slc51a	Bile secretion	mmu04976	-2.048676955
ENSMUSG00000020593	Lpin1	Alcoholic liver disease	mmu04936	-2.202453525
ENSMUSG00000026839	Upp2	Drug metabolism - other enzymes	mmu00983	-2.669502343
