# Supplementary figures and images for: Hepatoprotective Effects of Citri reticulatae Pericarpium and Chaenomelese speciosa (Sweet) Nakai Extracts in Alcohol-Related Liver Injury: Modulation of Oxidative Stress, Lipid Metabolism, and Gut Microbiota
Source: Antioxidants (Basel). 2025 Mar 14;14(3):343. doi: 10.3390/antiox14030343 (PMC11939523; doi:10.3390/antiox14030343)

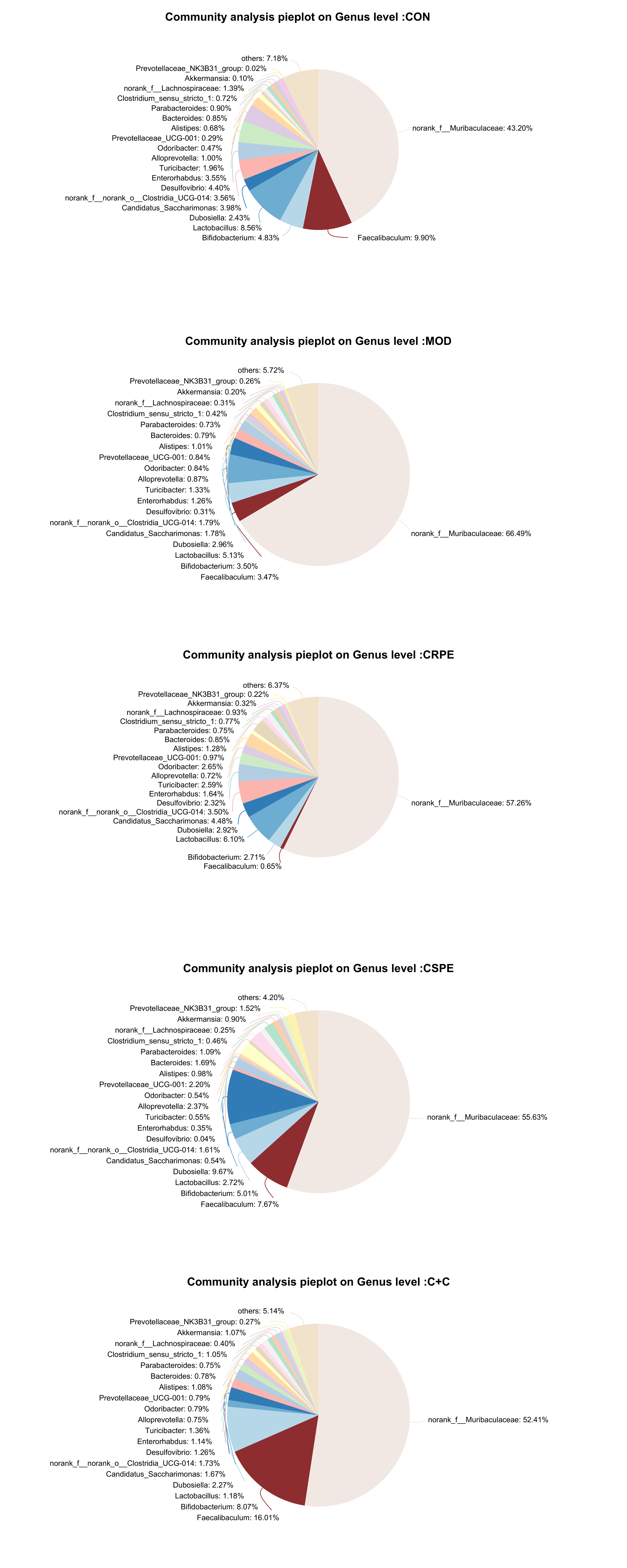

Supplement: Supplementary file 1 [file antioxidants-14-00343-s001.zip › Figure S1.png]

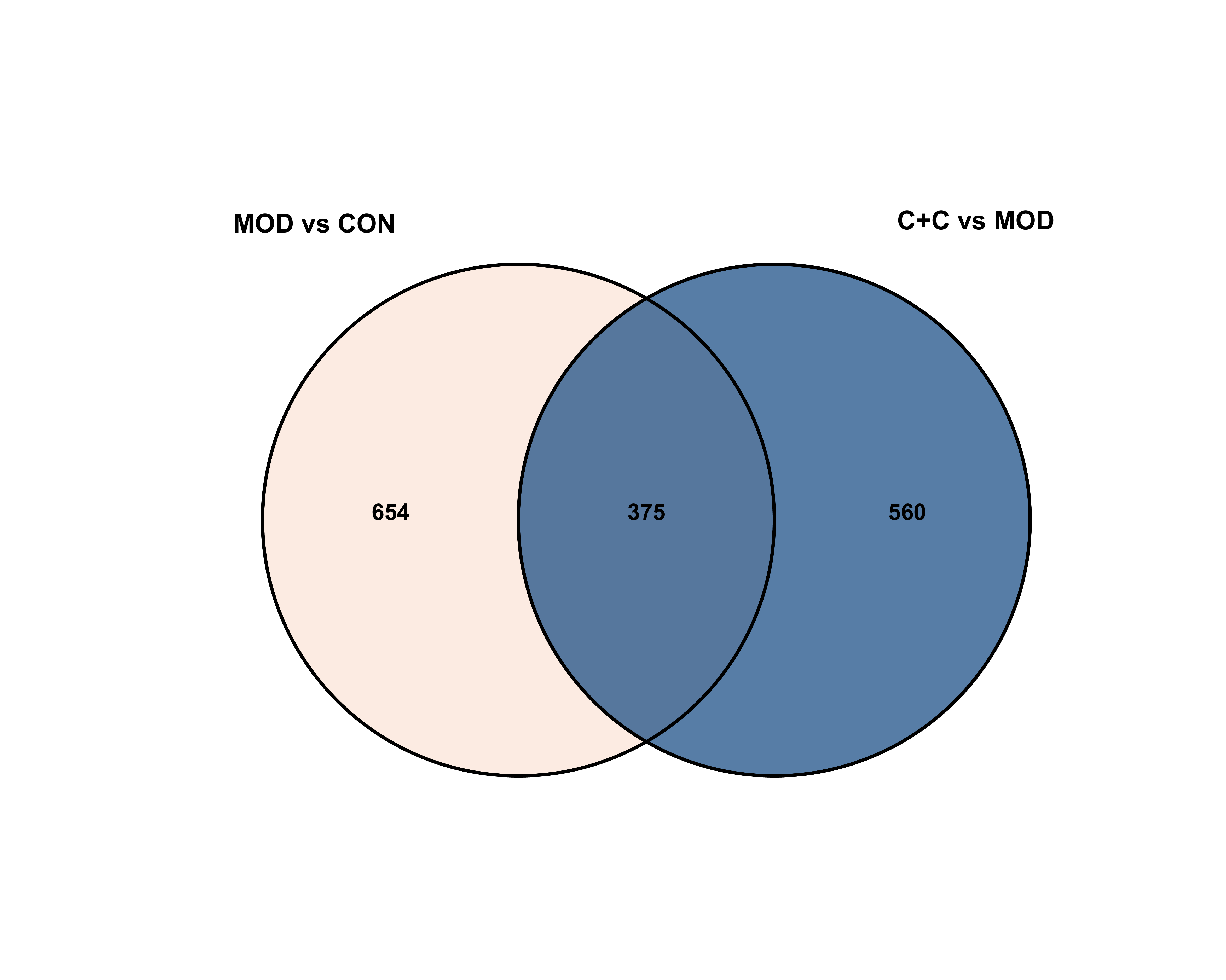

Supplement: Supplementary file 1 [file antioxidants-14-00343-s001.zip › Figure S2.png]
